# Cytotoxic and Anti-HSV-1 Effects of Caulerpin Derivatives

**DOI:** 10.3390/molecules29163859

**Published:** 2024-08-15

**Authors:** Gisely Maria Freire Abílio, Cicera Janaine Camilo, Henrique Douglas Melo Coutinho, José Galberto Martins da Costa, Lindomar José Pena, Abelardo Silva-Júnior, Yuri Mangueira do Nascimento, José Maria Barbosa-Filho, Bárbara Viviana de Oliveira Santos, Kristerson Reinaldo de Luna Freire

**Affiliations:** 1 Department of Physiology and Pathology, Federal University of Paraíba, João Pessoa 58051-900, PB, Brazil; gisely.abilio@academico.ufpb.br; 2 Department of Biological Chemistry, Regional University of Cariri, Crato 63105-010, CE, Brazil; janainecamilo@hotmail.com (C.J.C.);; 3 Oswaldo Cruz Foundation, Aggeu Magalhães Research Center, Recife 50740-465, PE, Brazil; 4 Institute of Biological and Health Sciences, Federal University of Alagoas, Maceió 57072-900, AL, Brazil; 5 Postgraduate Program in Natural and Synthetic Products Bioactive, Health Sciences Center, Federal University of Paraiba, João Pessoa 58051-900, PB, Brazil; 6 Graduate Program in Development and Technological Innovation in Medicines, Federal University of Campina Grande, Cajazeiras 58900-000, PB, Brazil; 7 Department of Cell and Molecular Biology, Biotechnology Center, Federal University of Paraíba, João Pessoa 58051-900, PB, Brazil

**Keywords:** *Caulerpa racemosa*, caulerpin, indolic derivatives, Vero cells, HSV-1

## Abstract

Marine organisms represent a potential source of secondary metabolites with various therapeutic properties. However, the pharmaceutical industry still needs to explore the algological resource. The species *Caulerpa lamouroux* Forssk presents confirmed biological activities associated with its major compound caulerpin, such as antinociceptive, spasmolytic, antiviral, antimicrobial, insecticidal, and cytotoxic. Considering that caulerpin is still limited, such as low solubility or chemical instability, it was subjected to a structural modifications test to establish which molecular regions could accept structural modification and to elucidate the cytotoxic bioactive structure in Vero cells (African green monkey kidney cells, *Cercopithecus aethiops*; ATCC, Manassas, VA, USA) and antiviral to *Herpes simplex* virus type 1. Substitution reactions in the *N*-indolic position with mono- and di-substituted alkyl, benzyl, allyl, propargyl, and ethyl acetate groups were performed, in addition to conversion to their acidic derivatives. The obtained analogs were submitted to cytotoxicity and antiviral activity screening against *Herpes simplex* virus type 1 by the tetrazolium microculture method. From the semi-synthesis, 14 analogs were obtained, and 12 are new. The cytotoxicity assay showed that caulerpin acid and *N*-ethyl-substituted acid presented cytotoxic concentrations referring to 50% of the maximum effect of 1035.0 µM and 1004.0 µM, respectively, values significantly higher than caulerpin. The antiviral screening of the analogs revealed that the *N*-substituted acids with methyl and ethyl groups inhibited *Herpes simplex* virus type 1-induced cytotoxicity by levels similar to the positive control acyclovir.

## 1. Introduction

Drug discovery for various therapeutic areas, especially cancer, infectious diseases, vascular diseases, and multiple sclerosis, for example, has expanded in recent years in proportion to investigations of natural products and their semisynthetic derivatives, which arguably play a key role for sources of new candidates for these drugs [1]. A good action plan for preparing derivatives is to enhance selectivity and therapeutic action arising from the promotion of physicochemical and pharmacokinetic activities and create patentable compounds. Thus, causing increased lipophilicity and promoting the insertion of atoms or groups of atoms into the chemical structures of natural products are excellent examples of changes that improve their biological activity [2].

In recent years, the number of organic compounds isolated from marine sources has been surprising due to industry research aiming to develop new drugs with therapeutic properties from the sea. In particular, seaweeds, whose commercial production has increased rapidly in recent decades, either by harvesting natural resources or by cultivation, and whose application is considered environmentally friendly, healthy, and sustainable for humans because of their many compounds that can be used as foods, cosmetics, medicines, and pharmaceuticals, can be applied in aquaculture and agriculture [3].

Alkaloids form a special class of secondary metabolites, grouped into heterocyclic and non-heterocyclic compounds based on the nitrogen atom position in their chemical structure. Most alkaloids are pharmacologically active or poisonous in excessive doses; they exhibit multiple biological activities, such as antitumor, antimicrobial, anticholinergic, antihypertensive, antidepressant, anti-inflammatory, and anti-ulcer, among others [4]. The literature mentions four alkaloid drugs of marine origin in clinical use, such as anticancer [ara-C (Cytarabine^®^) and trabectedin (Yondelis^®^)], antiviral [ara-A (Vidarabine^®^)], and neuropathic analgesic [ziconotide (Prialt^®^)] [5]. 

In this context, studies with *Caulerpa lamouroux* species proved the anti-inflammatory activity of the methanolic extract of *Caulerpa mexicana* in vitro and in vivo [6,7] and the anti-inflammatory [8] and ulcerative colitis activities of the methanolic extract of *Caulerpa racemosa* [7]. They described the antinociceptive [8], spasmolytic [9,10], and antiviral activities against the *Herpes simplex* virus type 1 (HVS-1) [11] of the bis-indolic alkaloid, caulerpin (**1**), the major component of these species. Other authors have described the antimicrobial, insecticidal, and cytotoxic activities of extracts of the genus *Caulerpa* [12,13].

Given the variety of pharmacological properties presented by caulerpin (1), Canché Chay et al. [14] reported the synthesis of this natural product, starting from solutions of indole molecules and with higher yields than observed in extraction and purification processes of natural products.

Thus, recognizing the importance of the genus *Caulerpa* in the production of chemical constituents of the most varied classes with pharmacological potential, this study proposed performing structural modifications on the major component of *Caulerpa racemosa*, caulerpin (**1**), aiming to establish which molecular regions will be able to tolerate structural modulation and to elucidate the bioactive conformation by evaluating its cytotoxic and antiviral effects on the HSV-1. 

## 2. Results and Discussion

### 2.1. Chemical Studies

**Figure 1:**
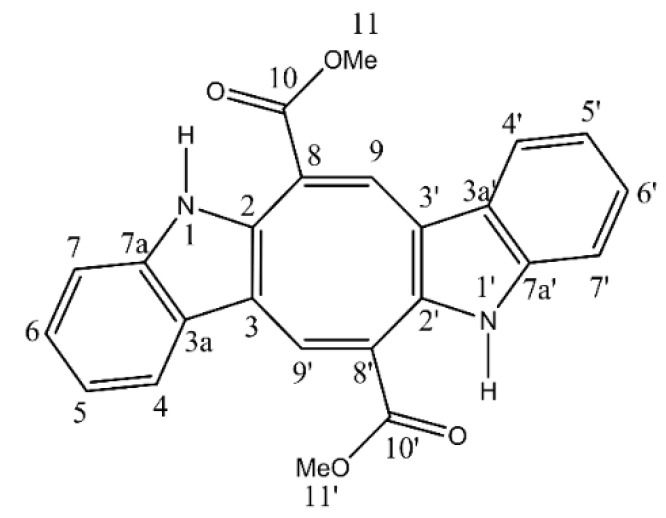
Structure of the bis-indolic alkaloid, caulerpin (**1**).

Caulerpin (**1**) was isolated from the extract of the green alga *C. racemosa* with a yield corresponding to 7% of the crude extract. The structure elucidation of the natural product was based mainly on the analysis of its IR, NMR ^1^H, and ^13^C spectra and in comparison with the literature data. The IR spectrum revealed bands at ν_max_ 3382 (N–H), 1687 (referring to C=O stretching of ester conjugated). The ^1^H NMR spectral data (Table 1) revealed four signals characteristic of *ortho*-disubstituted benzene: two doublets (δ 7.43, 1H and δ 7.30, 1H) and two triplets (δ 7.18, 1H and δ 7.09, 1H). The singlet observed at δ 9.21 suggests the presence of an indole nucleus, characteristic of the alkaloid class [15,16]. The presence of two singlets at δ 8.06 (1H) and δ 3.90 (3H) suggests the presence of cyclooctatetraene and methyl ester groups, respectively [17]. The ^13^C-APT NMR data (Table 1) confirm the presence of the benzene ring (δc 111.7, 118.4, 120.8, 123.5, hydrogenated aromatic carbons and δc 128.3, 137.8, non-hydrogenated aromatic carbons) and cyclooctatetraene (δc 142.9, hydrogenated aromatic carbon and δc 112.0, 133.0, 125.6, positive phase) in addition to the methyl ester group (δc 52.7, non-hydrogenated aromatic carbons) and the carbonyl group (δc 166.8) [18]. Given the results, it was possible to identify the compound from the *C. racemosa* extract as the majority alkaloid caulerpin (Figure 1), already isolated previously in several *Caulerpa* species [17].

The natural product caulerpin (**1**) comes from a family of bis-indolic alkaloids and has an extra eight-membered ring between two indolic rings directly incorporated with the carbonyl group. This alkaloid has several important biological activities already described in the literature. Macedo et al. [11] described **1** as an alternative drug to acyclovir^®^ during the treatment of HSV-1 infections by inhibiting the *alpha* and *beta* phases of the viral replication cycle. 

Several scientific reports mention the pharmacological properties of **1**, such as anti-inflammatory activity [7,8], antinociceptive [8], spasmolytic [9,10], and antituberculosis [14]. More recently, Esteves et al. [19] studied the antiviral activity of **1** against the *Chikungunya* virus, which showed a very significant and promising EC_50_ inhibitory effect of 0.8 μM, while virucidal activity proved very efficient in inhibiting nearly 90% of viral infectivity at 5 μM concentration.

Due to the promising biological activities of **1**, several analogs were proposed for elaboration, evaluation of their biological activities, and consequent study of structure-activity relationships.

**Scheme 1:**
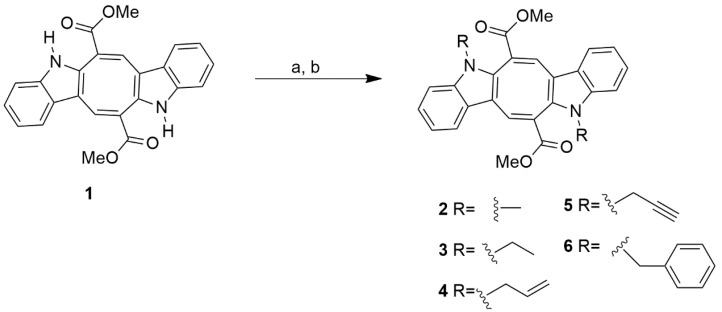
**2**–**6** derivatives of caulerpin (**1**). Reagents and conditions: (a) **2**: KOH, Me_2_SO_4_; MeOH, Acetone/room temperature, magnetic stirring; (b) **3**–**6**: KOH, RX (X = Cl or Br); DMF/room temperature, magnetic stirring.

The literature reports several examples of modified natural products with recognized pharmacological activity, such as nicotine (pyridine alkaloid); adrenaline, mescaline, morphine, and tubocurarine (tyrosine alkaloids); ephedrine and pseudoephedrine (phenylalanine alkaloids); vitamins B1, B2, and B5; some tropane alkaloids such as cocaine and atropine; and more complex products such as paclitaxel, testosterone, and progesterone [20]. The introduction of methyl (**2**) and ethyl (**3**) alkyl groups into the bis-indolic core of **1** increases lipophilicity, impacting permeability across biological membranes, which causes potentiation of its activity. In parallel, the insertion of unsaturated allyl (**4**) and propargyl (**5**) groups favors the adjustment with the respective receptors. In contrast, the insertion of aromatic rings (**6**) enables the enlargement of molecular dimensions, a property useful for receptor sites where there is a hydrophobic cavity liable to be occupied by the ring [21]. The structures of the analogs with substituents on the indolic nuclei are shown in Scheme 1.

**Scheme 2:**
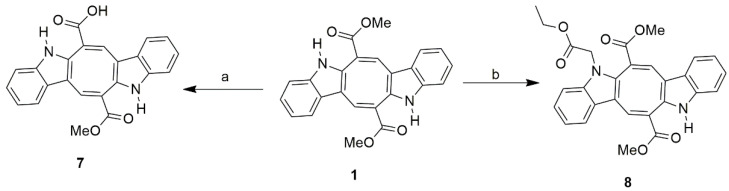
Derivatives **7** and **8** of caulerpin (**1**). Reagents and conditions: (a) **7**: KOH, 3,4,5-trihydroxy-benzyl chloride; DMF, 50 °C, magnetic stirring; (b) **8**: KOH, ethyl bromoacetate; DMF/50 °C, magnetic stirring.

Insertion of 3,4,5-trihydroxybenzyl groups into the indole nitrogen of caulerpin **1** using 3,4,5-trihydroxybenzyl chloride in DMF, according to the methodology of Zhao et al. [22], did not occur. Analyses of ^1^H and ^13^C NMR spectral data, including two-dimensional data, suggested that the reaction product would have the presence of two carbonyls, δC 168.4 (C-10), and 166.8 (C-10′), which correlate with H-9 and H-9′, respectively. However, only C-10′ demonstrates *^3^JCH* coupling with 3H-11′, suggesting the monoacid analog **7**, the result of a monohydrolysis of caulerpin **1** (Scheme 2). Furthermore, this monoindolic insertion behavior also occurred in the reaction of caulerpin **1** with ethyl bromoacetate [22], yielding analog **8** (Scheme 2).

**Scheme 3:**
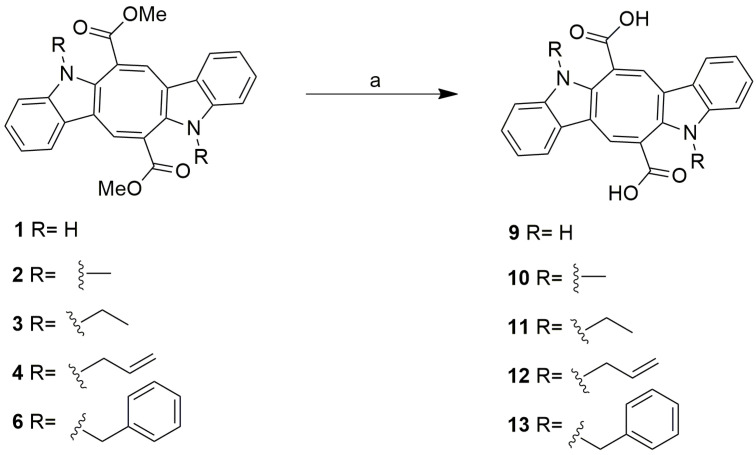
Acidic derivatives **9**–**13** from hydrolysis of **1**–**6**. Reagents and conditions: (a) KOH, Acetonitrile: water, 60 °C, magnetic stirring.

**Scheme 4:**
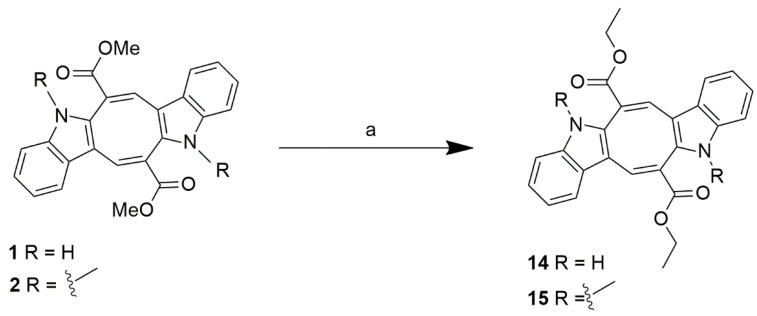
Transesterified derivatives **14** and **15**. Reagents and conditions: (a) KOH, EtOH; CH_2_Cl_2_, room temperature, magnetic stirring.

Prototype molecules with the introduction of acid groups in the chemical structure produce analogs with higher water solubility due to the ability of acids to form salts in vitro as the acidity in the structure increases. Usually, the most explored acid groups are carboxylic acid and sulfonic acid [21]. Aiming to obtain products with different polarities and, consequently, different pharmacological potentials, we proceeded with the production of analog **9** from the natural product **1**; and analogs **10**, **11**, **12**, and **13**, from the corresponding *N*-substituted **2**, **3**, **4**, and **6** (Scheme 3). All products were obtained by hydrolysis of the ester groups in a basic medium, with a nucleophilic addition–elimination reaction occurring at the ester carbonyl [23,24]. Two other analogs (**14** and **15**) are shown in Scheme 4, and were obtained to exchange the methyl groups of the esters for ethyl (**1** and **2**). The transesterification was carried out in order to amplify the lipophilic character [25]. Of the 15 molecules presented in this study, the analogs **2**, **3**, **4**, **5**, **6**, **7**, **8**, **10**, **11**, **12**, **13**, and **15** are reported for the first time in the literature.

### 2.2. Biological Studies

#### 2.2.1. Cytotoxic Effect on MTT

Cytotoxic analysis showed low activity for caulerpin (**1**), with a CC_50_ value of 687.9 ± 35.2 µM. In another study, caulerpin extracted from *C. peltata* also showed low cytotoxicity, showing 12% inhibition of cancer cell growth [26]. For analogs **2**, **4**, **5**, **12**, and **13**, lower CC_50_ values were observed than analog **1**, demonstrating greater cell growth inhibition (Table 2).

In another study that investigated the cytotoxicity of **1** against Vero cells, a CC50 of 1176 µM was verified, demonstrating that this compound has potential as a promising drug in human cells [11]. The difference in values obtained is related to the statistical methods used in the different works. The cytotoxicity of **1** was also evaluated in different colorectal cancer cells, showing an inhibitory effect on cell growth in the tested strains after 48 h of exposure, and the IC_50_ values ranged from 20 to 31 µM [27]. The effect of 1 in cancer cells may be associated with increased enzyme activity or decreased oxygen consumption by the cell [27]. 

Higher CC50 values were observed compared to analogs **9** and **11** and, consequently, lower cytotoxicity than for **1**. These results may be related to the hydrophilicity characteristics of the molecules, demonstrating that the insertion of lipophilic groups with different numbers of carbons, double bonds, and aromatic groups does not relate to the cytotoxic potency observed in Vero cells. 

In a cytotoxicity study performed with tri(1-alkyl-indol-3yl) methylene salts on human colon carcinoma HCT116 and leukemia K526 cells, it was observed that in both cell lines, the average CC_50_ decreases progressively with the increasing number of carbons up to the radical with five carbons, suggesting that the number of substituent carbons in the indolic nitrogen is related to higher cytotoxic potency [28].

Regarding the E_máx_ values of the analogs, it was evidenced that acids **12** and **13** presented a significantly lower effect (*p* < 0.05) than **1**, suggesting that the simultaneous presence of the acid group and the bulkier groups (allyl and benzyl) as substituents in the indole unit act to attenuate the cytotoxic efficiency. Differently, analogs **2**, **4**, **5**, **9**, **10**, and **11** provided E_máx_ values significantly higher than **1** (*p* < 0.05).

**Figure 2:**
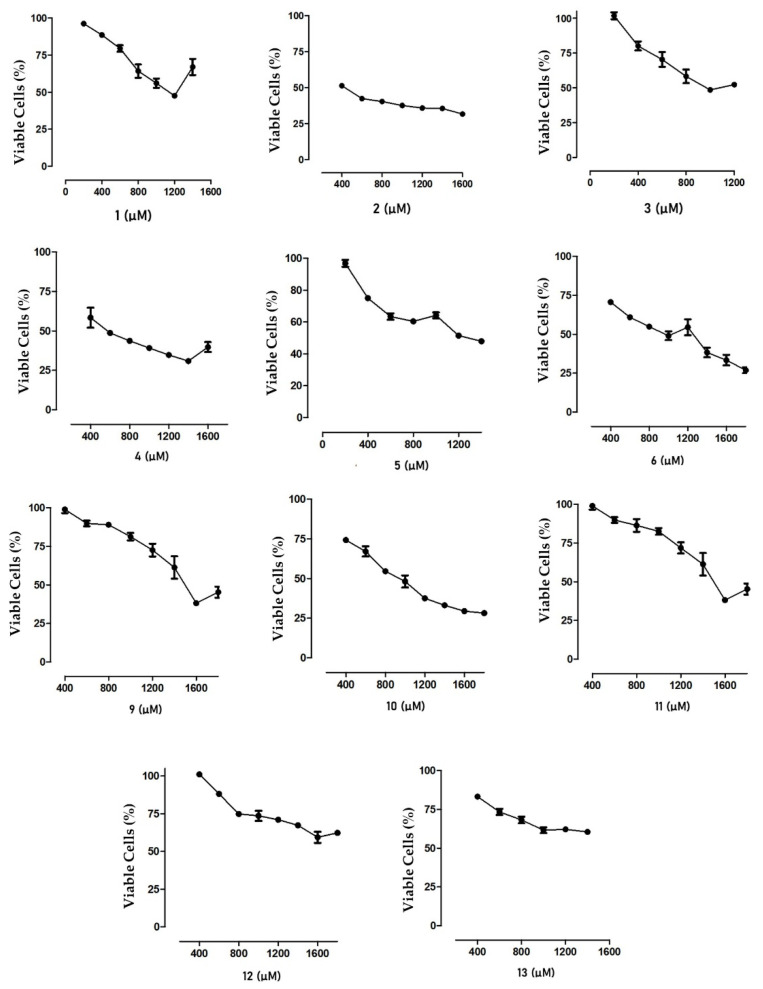
Graphs of the effect of **1** and its analogs on cell viability.

By analyzing the cell viability curves for each analog (Figure 2), it was possible to demonstrate that analogs **2** and **4** presented a maximum percentage of cell viability lower than 75%, showing high cytotoxicity compared to the other molecules. In general, the analogous compounds showed higher cytotoxicity than **1**. In most of them, cell growth inhibition was less than 50%, demonstrating viability for studying antiviral and antifungal activities with these compounds. 

#### 2.2.2. Antiviral Assay 

##### Concomitant Treatment of Infection

This assay sought to evaluate the samples’ ability to stop the infection at the same moment the cells were infected with the virus. As cell viability was less than 75%, analogs **2** and **4** were not tested in this assay.

**Figure 3:**
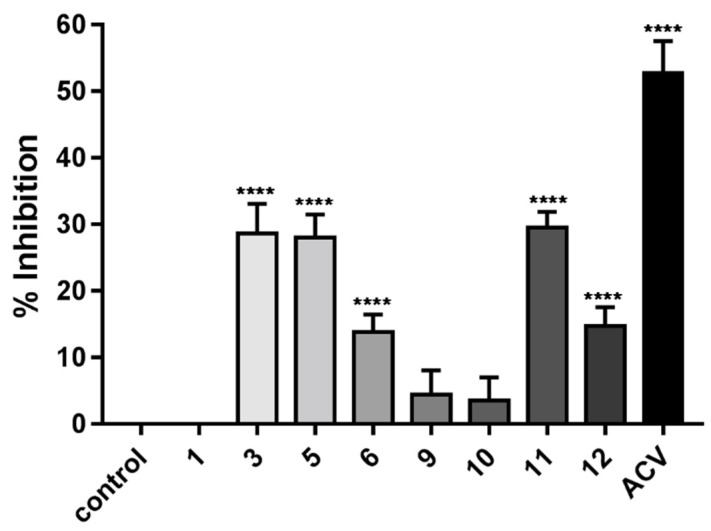
Percentage of HSV-1 inhibition by caulerpin analogs in Vero cells infected with HSV-1 (MOI 0.2). The cells were treated with the compound’s CC_20_ and further incubated at 37 °C in 5% CO_2_ for 72 h. Cell viability was assessed using the MTT method. Statistical analyses were performed with ANOVA and Tukey’s posttest (**** *p* < 0.0001 versus control). ACV—Acyclovir.

Treatment of cells with analogs **3**, **5**, **6**, **11**, and **12** resulted in a significant inhibition of HSV-1-induced cytotoxicity (*p* < 0.0001) when compared to the untreated cells (control) (Figure 3).

The results observed for analogs **3**, **5**, **6**, **11**, and **12** suggested that the introduction of methylene groups in chemical structures of prototype molecules increases their dimensions, as well as their lipophilicity, allowing for an increase in the potency of the biological properties. Only Acyclovir (ACV) obtained an inhibition percentage greater than 50% (52.79%).

Despite the numerical difference in the inhibition percentage values between acyclovir and molecules **11** and **12**, statistical analysis indicates that these new compounds exhibit efficacy similar to the antiviral agent acyclovir when the treatment occurs concurrently with the infection. Considering that acyclovir is one of the most widely used anti-HSV-1 drugs in clinical applications, molecules **11** and **12** represent an effective and safe alternative for treating the high rates of patients infected with this virus.

##### Post-Infection Treatment Assay

**Figure 4:**
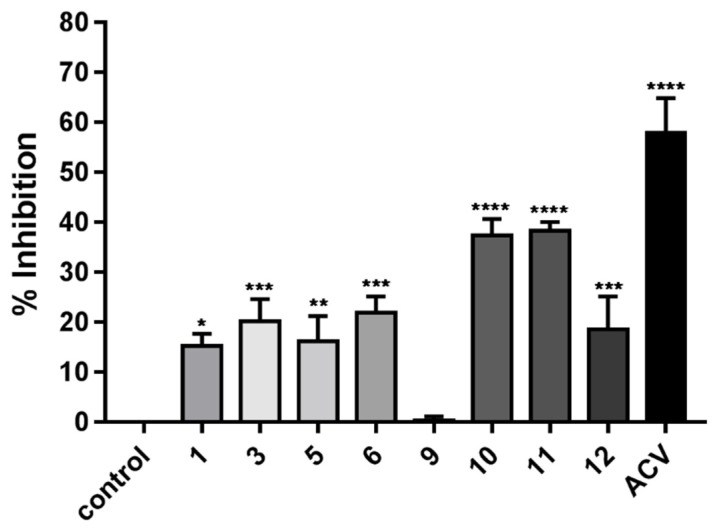
Inhibition of HSV-1-induced cytotoxicity by compound 1 and its analogs. Cells were infected with HSV-1 (MOI 0.2) for 1 h, washed, and then treated with the compound’s CC20. The plates were incubated at 37 °C in 5% CO_2_ for 72 h and cell viability was determined using the MTT method. Significant increase in the percentage of cell inhibition (ANOVA, with Dunnet’s posttest, * *p* < 0.05; ** *p* < 0.005; *** *p*< 0.0005; **** *p* < 0.0001): experimental groups versus control (infected and untreated).

In this assay, the ability of the compounds to block an HSV-1 infection already established in Vero cells was verified. The inhibition (%) of HSV-1-induced cytotoxicity after treatment with compound **1** and its analogs is shown in Figure 4. It was evidenced that treatment with **1** and analogs **3**, **5**, **6**, **10**, **11**, and **12** resulted in higher percentages of cellular inhibition compared to the control group, suggesting that the introduction of alkyl groups may enhance anti-HSV-1 activity in post-infection treatment. Previous studies have demonstrated the antiviral activity of seaweed extracts rich in 1 at concentrations ranging from 2.22 to 4.20 µg/mL [29,30]. 

The continuous use of available medications for HSV-1 treatment promotes the selection of resistant strains and their relative toxicities upon prolonged administrations. In this context, analogs **10** and **11** emerge as promising molecules in combating this virus by exhibiting antiviral effects statistically similar to acyclovir.

## 3. Materials and Methods

### 3.1. Chemistry

Spectra in the IR region were recorded on a Shimadzu IRprestige-21 Fourier Transform-Infrared Spectrophotometer. NMR spectra (^1^H, ^13^C, APT, COSY, HMQC, and HMBC) were recorded in CDCl_3_ and CD_3_OD (ACROS, Cambridge Isotope Laboratories (Tewksbury, MA, USA), Merck (Rahway, NJ, USA), or Sigma-Aldrich (St. Louis, MO, USA), with TMS as an internal standard) on a Bruker spectrometer (200 MHz (^1^H) and 500 MHz (^13^C)). Thin-layer chromatographies (analytical and preparative, TLC, and PTLC) were performed on pre-coated plates of 0.20 mm-thick silica gel 60 F_254_ (Macherey-Nagel, Dueren, Germany) and 1.0 mm-thick silica gel PF_254_7749 (Merck), respectively, and spots were visualized under a UV lamp (254 and 366 nm) and by spraying with a solution of perchloric acid-vanillin in EtOH, followed by heating. Chromatography columns were performed using Merck silica gel (Ø µm 63–200). All solvents and reagents were purchased from Vetec (Duque de Caxias, Brazil) and Merck-Sigma-Aldrich (Duque de Caxias, Brazil).

#### 3.1.1. Caulerpin Extraction and Isolation (**1**)

*Caulerpa racemosa* (Caulerpaceae) was collected in the city of Pitimbu, State of Paraíba, the northeastern region of Brazil, coordinates 7°07′31″ S; 34°49′25″, during high tides (−0.2 to 2.0). The species was identified by Prof. Dr. George Emmamuel Cavalcanti de Miranda of the Department of Molecular Biology/CCEN/UFPB, and an exsicata (# JPB 62814) was deposited in the Prof. Lauro Pires Xavier Herbarium at UFPB. The dried material was submitted to exhaustive extraction with EtOH, followed by its concentration in a rotary evaporator, obtaining the respective crude extract. A portion (30 g) of the crude extract of *C. racemosa* was submitted to open-column adsorption chromatography (CC) in silica gel (Merck or Vetec; Ø µm 63–200) and solvents hexane and CH_2_Cl_2_, with elution orders of hexane, hexane: CH_2_Cl_2_ (1:1), hexane: CH_2_Cl_2_ (8:2), and CH_2_Cl_2_. The fractions were concentrated at a rotary evaporator and pooled according to TLC analysis. This procedure provided 2.1 g (7%) of pure caulerpin (**1**) in the form of a red solid, named (6*E*,13*E*)-dimethyl-5,12-dihydrocycloocta[1,2b:5,6-b′]diindol-6,13-dicarboxylate IR (KBr) ν_max_/cm^−1^: 3382, 3032, 3054, 2997, 2952, 2927, 2852, 1687, 1630, 1612, 1459, 1443, 1416, 1323, 1265, 1202, 1176, 1056, 767, 730, 613, 596. ^1^H NMR (200 MHz, CDCl_3_, ppm) δ: 9,21 (s, 1H); 8,06 (s, 1H); 7.43 (d, 1H); 7.30 (d, 1H); 7.18 (t, 1H); 7.09 (t, 1H); 3.90 (s, 3H). ^13^C NMR (50 MHz, CDCl_3_, ppm) δ: 166.8 (C-10/10′); 142.9 (C-9/9′); 137.8 (C-7a/7a′); 133.0 (C-2/2′); 128,3 (C-3a/3a′); 125.6 (C-8/8′); 123.5 (C-6/6′); 120.8 (C-5/5′); 118.4 (C-4/4′); 112.6 (C-3/3′); 111.7(C-7/7′); 52.7 (C-11/11′).

#### 3.1.2. Derivatives Preparation

*(6E,13E)-Dimethyl-5,12-dimethyl-5,12 dihydrocycloocta[1,2 b: 5, 6-b′]diindol-6,13-dicarboxylate)* (**2**). KOH (29.17mg, 0.52 mmol) was added to a solution of **1** (80 mg, 0.2 mmol) in MeOH (10 mL) at room temperature. The MeOH was then distilled off, and acetone (10 mL) and (Me)_2_SO_4_ (0.06 mL, 0.6 mmol) were added to the reaction medium [31]. After 3 h, the solvent was evaporated, and liquid–liquid partitioning was performed using H_2_O (20 mL) and CH_2_Cl_2_ (2 × 20 mL). The organic fraction was dried, and PTLC was performed on silica gel (hexane: EtOAc, 8:2), yielding 43.2 mg of pure analog **2** (54%). Appearance: orange-red crystals; solubility: CH_2_Cl_2_; molecular formula: C_26_H_22_N_2_O_4_; molar mass: 426.46 g/mol. IR (KBr) ν_max_/cm^−1^: 3437, 3058, 2992, 2948, 1718, 1708, 1631, 1550, 1466, 1434, 1384, 1318, 1253, 1227, 1203, 1067, 1041, 908, 845, 734, 580. ^1^H NMR (200 MHz, CD_3_OD, ppm) δ: 8.49 (s, 1H); 7.54 (d, 1H); 7.23 (d, 1H); 7.16 (t, 1H); 7.12 (t, 1H); 3.81 (s, 3H); 3.46 (s, 3H). ^13^C NMR (50 MHz, CD_3_OD, ppm) δ: 166.4 (C-10/10′); 144.6 (C-9/9′); 138.7 (C-7a/7a′); 134.6 (C-2/2′); 126.6 (C-3a/3a′); 125.4 (C-8/8′); 123.0 (C-6/6′); 120.5 (C-5/5′); 118.8 (C-4/4′); 112.9 (C-3/3′); 52.5 (C-11/11′); 30.9 (C-12/12′).

##### Derivatives **3**–**8**, Insertion of Groups at Indolic Nitrogen

A mixture of a solution of **1** (40 mg, 0.1 mmol) in DMF (2 mL) with KOH (23 mg, 0.4 mmol) was added to each of the corresponding halides: ethyl bromide (0.3 mmol, 0.022 mL); allyl bromide (0.3 mmol, 0.026 mL); propargyl bromide (0.3 mmol, 0.026 mL); benzyl chloride (0.3 mmol, 0.035 mL); 3,4,5-trimethoxy-benzyl (0.3 mmol, 65 mg); and ethyl bromoacetate (0.3 mmol, 0.033 mL). Each mixture was stirred for 2 h at 50 °C, and after solvent evaporation, a liquid–liquid partition was performed using H_2_O (20 mL) and EtOAc (2 × 20 mL). The organic fractions were dried and PTLC was performed on silica gel (hexane: EtOAc, 8:2), yielding the corresponding purified derivatives [22].

*(6E,13E)-Dimethyl-5,12-diethyl-5,12 dihydrocycloocta[1,2 b: 5,6-b′]diindole-6,13-dicarboxylate* (**3**). Yield: (32.7 mg) 81,5%; aspect: yellow crystals; solubility: CH_2_Cl_2_; molecular formula: C_28_H_26_N_2_O_4_; molar mass: 454.52 g/mol. IR (KBr) ν_max_/cm^−1^: 3419, 3048, 3015, 2996, 2948, 2932, 2890, 1705, 1619, 1551, 1465, 1430, 1319, 1243, 1193, 1066, 1044, 917, 765, 740, 690. ^1^H NMR (200 MHz, CD_3_OD, ppm) δ: 8.44 (s, 1H); 7.51 (d, 1H); 7.26 (d, 1H); 7.19 (t, 1H); 7.10 (t, 1H); 4.01 (m, 1H); 3.80 (m,1H); 3.80 (s, 3H); 1.17 (t, 3H). ^13^C NMR (50 MHz, CD_3_OD, ppm) δ: 166.8 (C-10/10′); 144.3 (C-9/9′); 137.5 (C-7a/7a′); 134.0 (C-2/2′); 126.9 (c-3a/3a′); 126.1 (C-8/8′); 122.7 (C-6/6′); 120.4 (C-5/5′); 118.8 (C-4/4′); 113.2 (C-3/3′); 110.5 (C-7/7′); 52.6 (C-11/11′); 39.2 (C-12/12′); 14.8 (C-13/13′).
*(6E,13E)-Dimethyl-5,12-diethyl-5,12 dihydrocycloocta[1,2 b: 5,6-b′]diindol-6,13-dicarboxylate* (**4**). Yield: (33.0 mg) 82.5%; aspect: yellow crystals; solubility: CH_2_Cl_2_; molecular formula: C_30_H_26_N_2_O_4_; molar mass: 478.54 g/mol. IR (KBr) ν_max_/cm^−1^: 3416, 3051, 3000, 2948, 2927, 1715, 1702, 1619, 1555, 1461, 1371, 1312, 1259, 1234, 1191, 1065, 1037, 917, 750, 739, 724. ^1^H NMR (200 MHz, CD_3_OD, ppm) δ: 8.44 (s, 1H); 7.49 (d, 1H); 7.23 (d, 1H); 7.20 (t, 1H); 7.12 (t, 1H); 5.73 (m, 1H); 4.93 (t, 2H); 4.47 (ddd, 2H); 3.77 (s, 3H). ^13^C NMR (50 MHz, CD_3_OD, ppm) δ: 166.5 (C-10/10′); 144.5 (C-9/9′); 137.9 (C-7a/7a′); 134.1 (C-2/2′); 132.9 (C-13/13′); 126.6 (C-3a/3a′); 126.0 (C-8/8′); 122.9 (C-6/6′); 120.4 (C-5/5′); 118.4 (C-4/4′); 117.1 (C-14/14′); 113.5 (C-3/3′); 110.6 (C-7/7′); 52.4 (C-11/11′); 47.1 (C-12/12′).
*(6E,13E)-Dimethyl-5,12-di(prop-2-yn-1-yl) 5,12-dihydrocycloocta[1,2b:5,6-b′]diindol-6,13 dicarboxylate* (**5**). Yield: (34.0 mg) 85%; aspect: yellow crystals; solubility: CH_2_Cl_2_; molecular formula: C_30_H_22_N_2_O_4_; molar mass: 474.51 g/mol. IR (KBr) ν_max_/cm^−1^: 3475, 3416, 3284, 3057, 2948, 2923, 2849, 2100 1711, 1624, 1550, 1462, 1433, 1388, 1333, 1315, 1266, 1240, 1194, 1071, 1043, 741, 662. ^1^H NMR (200 MHz, CD_3_OD, ppm) δ: 8.46 (s, 1H); 7.51 (d, 1H); 7.41 (d, 1H); 7.24 (t, 1H); 7.14 (t, 1H); 4.59 (m, 2H); 3.80 (s, 3H); 2.20 (s, 1H). ^13^C NMR (50 MHz, CD_3_OD, ppm) δ: 166.5 (C-10/10′); 145.6 (C-9/9′); 137.6 (C-7a/7a′); 133.6 (C-2/2′); 126.9 (C-3a/3a′); 125.9 (C-8/8′); 123.5 (C-6/6′); 120.6 (C-5/5′); 118.9 (C-4/4′); 114.2 (C-3/3′); 110.4 (C-7/7′); 77.7 (C-14/14′); 73.1 (C-13/13′); 52.1 (C-11/11′); 34.4 (C-12/12′).
*(6E,13E)-Dimethyl-5,12-dinzyl 5,12-dihydrocycloocta[1,2b:5,6-b′]diindol-6,13-dicarboxylate* (**6**). Yield: (39.7 mg) 99.25%; aspect: yellow crystals; solubility: CH_2_Cl_2_; molecular formula: C_38_H_30_N_2_O_4_; molar mass: 578.66 g/mol. IR (KBr) ν_max_/cm^−1^: 3474, 3415, 3058, 3032, 2948, 1691, 1617, 1550, 1463, 1428, 1263, 1244, 1190, 1069, 1051, 914, 768, 737, 707, 668, 613. ^1^H NMR (200 MHz, CD_3_OD, ppm) δ: 8.45 (s, 1H); 7.35 (d, 1H); 7.31 (d, 1H); 7.05 (t, 1H); 6.95 (t, 1H); 5.10 (m, 2H); 3.66 (s, 3H). ^13^C NMR (50 MHz, CD_3_OD, ppm) δ: 166.3 (10/10′); 144.9 (C-9/9′); 138.0 (C-13); 136.9 (C-7a/7a′); 134.4 (C-2/2′); 128.5 (C-*orto*); 127.3 (C-*meta*); 126.8 (C-3a/3a′); 126.6 (C-8/8′); 126.2 (C-*para*); 123.2 (C-6/6′); 120.6 (C-5/5′); 119.4 (C-4/4′); 114.1 (C-3/3′); 110.6 (C-7/7′); 52.4 (C-11/11′); 48.1(C-12).
*(6E,13E)-13-(Metoxicarbonyl) 5,12-dihidrocicloocta[1,2b:5,6-b′]diindol-6-ácidocarboxílico* (**7**). Yield: (12.9 mg) 32.25%; aspect: dark crystals; solubility: MeOH; molecular formula: C_23_H_16_N_2_O_4_; molar mass: 384.38g/mol. ^1^H NMR (200 MHz, CD_3_OD, ppm) δ: 8.21 (s, 1H); 8.17 (s, 1H); 7.41 (d, 1H); 7.30 (d, 1H); 7.10 (t, 1H); 7.02 (t, 1H); 4.81 (s); 3.89 (s, 3H). ^13^C NMR (50 MHz, CD_3_OD, ppm) δ: 168.4 (C-10); 166.8 (C-10′); 143.9 (C-9′); 139.5 (C-7a); 139.4 (C-7a′); 135.3 (C-2); 134.1 (C-2′); 129.3 (C-3a); 129.2 (C-3a′); 129.9 (C-8′); 126.9 (C-8); 123.8 (C-6′); 121.3 (C-5); 121.1 (C-5′); 118.9 (C-4); 118.7 (C-4′); 113.5 (C-3); 112.9 (C-3′); 112.7 (C-7); 112.6 (C-7′); 53.0(C-11′).
*(6E,13E)-Dimethyl-5-(2-oxybutyl) 5,12-dihydrocycloocta[1,2b:5,6-b′]diindol-6,13-dicarboxylate* (**8**). Yield: (5.47mg) 13.67%; aspect: yellow solid; solubility: CH_2_Cl_2_; molecular formula: C_28_H_24_N_2_O_4_; molar mass: 484.50 g/mol. ^1^H NMR (200 MHz, CD_3_OD, ppm) δ: 9.11 (s, 1H); 8.35 (s, 1H); 8.17 (s, 1H); 7.45 (d, 1H); 7.43 (d, 1H); 7.28 (d, 1H); 7.21 (d, 1H); 7.17 (t, 1H); 7.16 (t, 1H); 7.11 (t, 1); 7.08 (t, 1H); 4.65 (m, 1H); 4.48 (m, 1H); 4.00 (m, 2H); 3.88 (s, 3H); 3.76 (s, 3H); 0.97 (t, 3H). ^13^C NMR (50 MHz, CD_3_OD, ppm) δ: 167.9 (C-13); 166.8 (C-10); 165.9 (C-10′); 146.3 (C-9); 141.5 (C-9′); 138.9 (C-7a); 137.3 (C-7a′); 134.1 (C-2); 132.2 (C-2′); 127.6 (C-3a); 126.8 (C-3a′); 126.4 (C-8); 124.8 (C-8′); 123.2 (C-6′); 120.8 (C-5); 120.6 (C-5′); 118.8 (C-4); 118.4 (C-4′); 113.6 (C-3); 113.3 (C-3′); 111.5 (C-7); 110.1 (C-7′); 61.5 (C-14); 52.6 (C-11); 52.3 (C-11′); 45.9 (C-12); 13.8 (C-15).

##### Derivatives **9**–**13**, Hydrolysis of Ester Groups

The acids **1**, **2**, **3**, **4**, and **6** were obtained using the methodology proposed by Amarante et al. (2011). Solutions of **1** (40 mg, 0.1 mmol), **2** (40 mg, 0.094 mmol), **3** (40 mg, 0.091 mmol), **4** (40 mg, 0.092 mmol), and **6** (40 mg, 0.081 mmol) were mixed in ACN: H_2_O (8:2; 10 mL) with KOH: 80 mg, 1.43 mmol for **1**, **2**, **3**, and **4** and 72.37 mg, 1.29 mmol for **6**. Each mixture was placed in reflux for 2 h at 70 °C, and after the solvent evaporation, we proceeded with the liquid–liquid partitioning using HCl (1 mol·L^−1^, 20 mL), followed by treatment with EtOAc (2 × 20 mL). The organic fractions were dried and subjected to PTLC on silica gel (MeOH: CH_2_Cl_2_ 1:1), yielding the corresponding purified derivatives.

*(6E,13E)-5,12-Dihydrocycloocta[1,2b:5,6-b′]diindol-6,13-dicarboxylic acid* (**9**). Yield: (31.6 mg) 79%; aspect: black solid; solubility: MeOH; molecular formula: C_22_H_14_N_2_O_4_; molar mass: 370.36 g/mol. IR (KBr) ν_max_/cm^−1^: 3408, 3058, 3035, 2968, 2924, 2620, 2503, 1650, 1611, 1406, 1385, 1259, 1238, 1150, 7017, 755, 723, 611, 548. ^1^H NMR (200 MHz, CD_3_OD, ppm) δ: 8.21 (s, 1H); 7.37 (d, 1H); 7.28 (d, 1H); 7.08 (t, 1H); 7.01 (t, 1H); and 4.90 (s). ^13^C NMR (50 MHz, CD_3_OD, ppm) δ: 169.3 (C-10/10′); 143.7 (C-9/9′); 139.1 (C-7a/7a′); 134.3 (C-2/2′); 128.9 (C-3a/3a′); 127.3 (C-8/8′); 123.6 (C-6/6′); 120.8 (C-5/5′); 118.4 (C-4/4′); 112.7 (C-3/3′); 112.3 (C-7/7′).
*(6E,13E)-5,12-Dimethyl-5-dihydrocycloocta[1,2b:5,6-b’]diindol-6,13-dicarboxylic acid* (**10**). Yield: (23.5 mg) 58,8%; aspect: yellow crystals; solubility: MeOH; molecular formula: C_24_H_18_N_2_O_4_; molar mass: 398.41 g/mol. IR (KBr) ν_max_/cm^−1^: 3416, 3050, 2940,1885, 1880, 1624, 1554, 1488, 1376, 1317, 1250, 1221, 1220,1129, 1110, 930, 844, 742, 730, 701, 662. ^1^H NMR (200 MHz, CD_3_OD, ppm) δ: 8.33 (s, 1H); 7.49 (d, 1H); 7.40 (d, 1H); 7.20 (t, 1H); 7.09 (t, 1H); 3.45 (s). ^13^C NMR (50 MHz, CD_3_OD, ppm) δ: 175.9 (C-10/10′); 152.0 (C-9/9′); 147.5 (C-7a/7a′); 144.3 (C-2/2′); 135.9 (C-3a/3a′); 135.3 (C-8/8′); 132.1 (C-6/6′); 129.7 (C-5/5′); 127.8 (C-4/4′); 121.5 (C2-3/3′); 119.9 (C-7/7′); 40.2 (C-12/12′).
*(6E,13E)-5,12-Diethyl-5,12-dihydrocycloocta[1,2b:5,6-b′]diindol-6,13-dicarboxylic acid* (**11**). Yield: (30.4 mg) 76%; aspect: yellow crystals; solubility: MeOH; molecular formula: C_26_H_22_N_2_O_4_; molar mass: 426.46 g/mol. IR (KBr) ν_max_/cm^−1^: 3411, 3125, 3057, 2975, 2933, 2595, 1704, 1676, 1629, 1618, 1550, 1460, 1383, 1371, 1355, 1232, 1212, 1132, 747, 730, 700, 687, 536. ^1^H NMR (200 MHz, CD_3_OD, ppm) δ: 8.41 (s, 1H); 7.45 (d, 1H); 7.28 (d, 1H); 7.15 (t, 1H); 7.01 (t, 1H); 4.95 (s); 4.06(m, 1H); 3.87 (m, 1H); 1.07 (t, 3H). ^13^C NMR (50 MHz, CD_3_OD, ppm) δ: 168.2 (C-10/10′); 144.1 (C-9/9′); 136.7 (C-7a/7a′); 135.3 (C-2/2′); 127.9 (C-3a/3a′); 126.6 (C-8/8′); 123.6 (C-6/6′); 121.1 (5/5′); 119.1 (4/4′); 114.4 (C-3/3′); 110.9 (C-7/7′); 40.1 (C-12/12′); 15.1 (C-13/13′).
*(6E,13E)-5,12-Diallyl-5,12-dihydrocycloocta[1,2b:5,6-b′]diindol-6,13-dicarboxylic acid* (**12**). Yield: (25.9 mg) 64.86%; aspect: yellow crystals; solubility: MeOH; molecular formula: C_28_H_22_N_2_O_4_; molar mass: 450.49 g/mol. IR (KBr) ν_max_/cm^−1^: 3468, 3412, 3077, 3056, 2980, 2927, 1690, 1677, 1619, 1461, 1384, 1265, 1237, 1199, 1014, 990, 921, 833, 822, 738, 662. ^1^H NMR (200 MHz, CD_3_OD, ppm) δ: 8.40 (s, 1H); 7.43 (d, 1H); 7.26 (d, 1H); 7.15 (t, 1H); 7.07 (t, 1H); 5.70 (m, 1H); 4.93 (s); 4.57 (m, 2H). ^13^C NMR (50 MHz, CD_3_OD, ppm) δ: 169.2 (C-10/10′); 144.5 (C-9/9′); 139.3 (C-7a/7a′); 135.6 (C-2/2′); 134.4 (C-13/13′); 128.4 (3a/3a′); 127.8 (C-8/8′); 123.8 (C-6/6′); 121.3 (C-5/5′); 119.7 (C-4/4′); 116.5 (C-14/14′); 114.9 (C-3/3′); 111.5 (C-7/7′); 47.4 (C-12/12′).
*(6E,13E)-5,12-Dibenzyl-5,12-dihydrocycloocta[1,2 b: 5,6-b′]diindol-6,13-dicarboxylic acid* (**13**). Yield: (20.3 mg) 50.63%; aspect: yellow crystals; solubility: MeOH; molecular formula: C_36_H_26_N_2_O_4_; molar mass: 550.60 g/mol. IR (KBr) ν_max_/cm^−1^: 3415, 3086, 3060, 3028, 2943, 2865, 1701, 1663, 1619, 1560, 1462, 1370, 1333, 1261, 1200, 1185, 1027, 926, 831, 744, 727, 692, 607, 581. ^1^H NMR (200 MHz, CD_3_OD, ppm) δ: 8.42 (s, 1H); 7.11 (m); 6.73 (t, 1H); 6.56 (d, 2H); 6.40 (t, 2H); 5.22 (t, 2H); 3.66 (s). ^13^C NMR (50 MHz, CD_3_OD, ppm) δ: 167.8 (C-10/10′); 144.9 (C-9/9′); 139.1 (C-13/13′); 138.6 (C-7a/7a′); 135.9 (C-2/2′); 129.1 (C-*orto*); 128.2 (C-8/8′); 128.8 (C-3a/3a′); 127.8 (C-*meta*); 126.9 (C-*para*); 123.8 (C-6/6′); 121.3 (C-5/5′); 119.8 (C-4/4′); 115.6 (C-3/3′); 111.4 (C-7/7′); 30.7 (C-12/12′).

##### Derivatives **14** and **15** Transesterified

Solutions of **1** (35 mg, 0.075 mmol) and **2** (46 mg, 0.075 mmol) in CH_2_Cl_2_ (2mL) were individually added to EtOH (100 µL, 1.72 mmol) under stirring for 30 min at room temperature. Afterward, KOH (11 mg, 0.20 mmol) was added, and the reaction mixture was elevated to 40 °C for 1h. Then, a liquid–liquid partition was performed using CH_3_CO_2_H (1 mol.L^−1^, 20 mL), followed by treatment with EtOAc (2 × 20 mL). The organic fractions were dried, and PTLC was performed on silica gel (Hexane: EtOAc, 8:2), yielding the corresponding purified derivatives.

*(6E,13E)-Diethyl 5,12-dihydrocycloocta[1,2b:5, 6-b′]diindol-6,13-dicarboxylate* (**14**). Yield: (11.6 mg) 33.14%; aspect: red crystals; solubility: CH_2_Cl_2_; molecular formula: C_26_H_22_N_2_O_4_; molar mass: 426.46 g/mol. ^1^H NMR (200 MHz, CD_3_OD, ppm) δ: 9.27 (s, 1H); 7.41 (d, 1H); 7.28 (d, 1H); 7.16 (t, 1H); 7.01 (t, 1H); 4.34 (q, 2H); 1.40 (t,3H). ^13^C NMR (50 MHz, CD_3_OD, ppm) δ: 161.4 (C-10/10′); 137.5 (C-9/9′); 132.8 (C-7a/7a′); 128.1 (C-2/2′); 123.7 (C-3a/3a′); 120.9 (C-8/8′); 118.6 (C-6/6′); 115.7 (C-5/5′); 112.9 (C-4/4′); 107.5 (C-3/3′); 106.6 (C-7/7′); 56.7 (C-11/11′); 9.4 (C-12/12′).
*(6E,13E)-Diethyl 5,12-dimethyl-5,12-dihydrocycloocta[1,2b:5,6-b′]diindol-6,13-dicarboxylate* (**15**). Yield: (19.3 mg) 42%; aspect: red crystals; solubility: CH_2_Cl_2_; molecular formula: C_28_H_26_N_2_O_4_; molar mass: 454.52 g/mol. ^1^H NMR (200 MHz, CD_3_OD, ppm) δ: 8.45 (s, 1H); 7.52 (d, 1H); 7.21 (d, 1H); 7.14 (d, 1H); 7.10 (d, 1H); 4.28 (m, 2H); 3.45 (s, 3H); 1.30 (t,3H). ^13^C NMR (50 MHz, CD_3_OD, ppm) δ: 165.9 (C-10/10′); 144.4 (C-9/9′); 138.7 (C-7a/7a′); 134.8 (C-2/2′); 126.7 (C-3a/3a′); 125.8 (C-8/8′); 122.9 (C-6/6′); 120.8 (C-5/5′); 119.1 (C-4/4′); 112.9 (C-3/3′); 109.9 (C-7/7′); 61.5 (C-11/11′); 31.0 (C-13/13′); 14.6 (C-12/12′).

### 3.2. Cytotoxicity

The method of Cheng et al. [32] was followed with modifications to evaluate the cytotoxicity of caulerpin analogs **1**, **2**, **3**, **4**, **5**, **7**, **8**, **9**, **10**, and **11** at concentrations ranging from 200 to 1800 µM. Vero cells (African green monkey kidney cells *Cercopithecus aethiops*; ATCC, Manassas, VA, USA) grown in Dulbecco’s modified medium (DMEM) supplemented with 5% fetal bovine serum (FBS), 0.1 µM HEPES, and 2.5 µg/mL gentamicin at 37 °C in 5% CO_2_ were used. Each test sample was diluted in DMEM containing 2% fetal bovine serum at six different concentrations. Vero cells were plated at a concentration of 2 × 10^4^ cells/well and incubated for 24–36 h. When the cells showed 90% confluence, the medium was removed from the wells, and the samples were added and incubated for 72 h. After this time, the medium was discarded and 20 μL of MTT solution was added to each well. The plates were incubated for four hours at 37 °C. Subsequently, the supernatant was removed, and 150 μL of DMSO was added to each well to dissolve the formazan crystals. The plates were shaken for 10 min, followed by absorbance reading in an ELISA microplate reader at a wavelength of 540 nm.

The MTT assay was validated by constructing a trend line using linear regression in the GraphPad Prism Version 6.01 program. Assays that presented R^2^ greater than 0.70 were considered appropriate. After the validation of the MTT assay, the CC_50_ and CC_20_ values of each tested analog were calculated.

### 3.3. Antiviral Screening

The antiviral screening tests were performed following the method of Cheng et al. [32], with modifications. For the anti-HSV-1 (herpes simplex virus type 1) activity of caulerpin analogs, Vero cells were used under the same conditions as described for the cytotoxicity test. The assay was divided into two steps:

#### 3.3.1. Treatment Concomitant to Infection

Cells were infected with HSV-1 (MOI 0.2) and treated with the compound’s CC_20_ (concentration toxic to 20% of cells). The plates were incubated at 37 °C in 5% CO_2_ for 72 h, and cell viability was assessed using the MTT method.

#### 3.3.2. Post-Infection Treatment

After removing the culture medium, the HSV-1 (MOI 0.2) was added to the wells containing cells for 1 h. After this time, the cells were washed, and the tested compounds and acyclovir were added to their CC_20_. The plates were incubated at 37 °C in 5% CO_2_ for 72 h and cell viability was assessed using the MTT method.

In both assays, wells containing only cell medium were used as controls for cell growth. For positive control, wells containing medium and virus were used, and wells containing medium, virus, and acyclovir were used as a negative control.

The inhibition ratio was calculated using the following equation.

$$\%\;Inhibition=\frac{\left(OD\;treatment-OD\;positivecontrol\right)}{\left(OD\;cell\;growth-OD\;positive\;control\right)}\times 100$$

where OD = Optical density (absorbance).

## 4. Conclusions

Caulerpin (**1**) enabled the semi-synthesis of 14 analogs, including 12 unpublished in the literature. The identified molecules comprise five *N*-bisubstituted esters with methyl, ethyl, allyl, propargyl, and benzyl groups and one *N*-monosubstituted ester analog with ethyl acetate group.

The natural product **1** and its *N*-substituted ester analogs served as starting material for obtaining their respective acids, totaling the semi-synthesis of caulerpin acid and four other *N*-bisubstituted acid analogs by the respective methyl, ethyl, allyl, benzyl, and monoacid groups of **1**. Transesterification reactions yielded the -*O*-ethyl and *N*-methyl *O*-ethyl analogs.

The evaluation of the cytotoxic potential of the analogs of 1 through MTT analysis in Vero cells showed that caulerpin acid and caulerpin acid *N*-ethyl showed lower cytotoxic potentials when compared to **1**. The antiviral screening assay in Vero cells showed that *N*-ethyl, *N*-propargyl, *N*-benzyl esters, and *N*-ethyl and *N*-allyl acids were able to promote greater viability of Vero cells infected with HSV-1 when compared to the untreated group and caulerpin (*p* < 0.05) in the pre-infection phase. The *N*-ethyl and *N*-allyl acid analogs exhibit efficacy similar to the antiviral agent acyclovir when the treatment occurs concurrently with the infection.

For the analysis of antiviral screening after infection, it was observed that caulerpin, *N*-ethyl esters, *N*-propargyl, *N*-benzyl, *N*-methyl, and *N*-ethyl and *N*-allyl acids showed significant percentages of viral inhibition (*p* < 0.05). Among these, the *N*-methyl and *N*-ethyl acid analogs emerge as promising molecules in combating this virus by exhibiting antiviral effects statistically similar to acyclovir.

The structural modifications that resulted in the production of caulerpin *N*-substituted by allyl and propargyl groups stand out among all the reactions proposed for having promoted potentiation of the antiviral effect in the pre-infection and post-infection phases when compared to caulerpin (*p* < 0.05).

## Figures and Tables

**Table 1:** Comparative NMR data of Caulerpin (**1**) with the literature data [17].

	Analyzed Compound		Caulerpin [17]	
Carbons	δ_H_	δ_C_	δ_H_	δ_C_
N	9.21 (s, 1H)	—	9.20 (s, 1H)	—
2/2′	—	133.0 (q)	—	132.8 (q)
3/3′	—	112.0 (q)	—	112.4 (q)
3a/3a′	—	128.3 (q)	—	128.1 (q)
4/4′	7.43 (d, 1H)	118.4 (CH)	7.41(d, 1H)	118.0 (CH)
5/5′	7.09 (t, 1H)	120.8 (CH)	7.07 (t, 1H)	120.7 (CH)
6/6′	7.18 (t, 1H)	123.5 (CH)	7.17 (t, 1H)	123.3 (CH)
7/7′	7.30 (d, 1H)	111.7 (CH)	7.29 (d, 1H)	111.5 (CH)
7a/7a′	—	137.8 (q)	—	137.7 (q)
8/8′	—	125.6 (q)	—	125.6 (q)
9/9′	8.06 (s, 1H)	142.9 (CH)	8.04 (s, 1H)	142.9 (CH)
10/10′	—	166.8 (q)	—	166.6 (q)
11/11′	3.90 (s, 3H)	52.7 (CH_3_)	3.88 (s, 3H)	52.6 (CH_3_)

**Table 2:** Absolute CC_50_ values of **1** and its analogs against Vero cells (ANOVA *p* < 0.05).

Samples	E_max_ (%)	CC_50_ (µM)
Analog 1	52.7 ± 1.8 ^b^	687.9 ± 35.2 ^B^
Analog 2	68.4 ± 0.2 ^c^	524.1 ± 19.3 ^B^
Analog 3	51.5 ± 0.9 ^b^	547.6 ± 38.8 ^B^
Analog 4	70.1 ± 1.1 ^c^	628.2 ± 69.8 ^B^
Analog 5	52.1 ± 0.9 ^b^	496.1 ± 16.8 ^B^
Analog 6	74.1 ± 1.8 ^c^	891.0 ± 87.8 ^B^
Analog 9	62.6 ± 0.7 ^c^	1035.0 ± 62.4 ^A^
Analog 10	71.8 ± 0.3 ^c^	678.7 ± 38.3 ^B^
Analog 11	62.7 ± 0.8 ^c^	1004.0 ± 41.6 ^A^
Analog 12	44.5 ± 1.3 ^a^	663.9 ± 55.3 ^B^
Analog 13	41.0 ± 1.2 ^a^	494.0 ± 51.4 ^B^

Different lowercase letters in the same column represent a significant difference between the analogs. Different capital letters in the same column represent a significant difference between the analogs.

## Data Availability

Data is contained within the article and Supplementary Materials.

## Supplementary Materials

The following supporting information can be downloaded at: https://www.mdpi.com/article/10.3390/molecules29163859/s1.
