# Supplementary material for: Cytotoxic and Anti-HSV-1 Effects of Caulerpin Derivatives
Source: Molecules. 2024 Aug 15;29(16):3859. doi: 10.3390/molecules29163859 (PMC11357404; doi:10.3390/molecules29163859)

Supplementary Materials

Cytotoxic and Anti-HSV-1 Effects of Caulerpin Derivatives

Gisely Maria Freire Abílio [1], Cicera Janaine Camilo [2], Henrique Douglas Melo Coutinho [2,*], José Galberto Martins da Costa [2], Lindomar José Pena [3], Abelardo Silva-Júnior [4], Yuri Mangueira do Nascimento [5], José Maria Barbosa-Filho [5], Bárbara Viviana de Oliveira Santos [6,*] and Kristerson Reinaldo de Luna Freire [7]

1 Department of Physiology and Pathology, Federal University of Paraíba, João Pessoa 58051-900, PB, Brazil; gisely.abilio@academico.ufpb.br
2 Department of Biological Chemistry, Regional University of Cariri, Crato 63105-010, CE, Brazil; janainecamilo@hotmail.com (C.J.C.); galberto.martins@gmail.com (J.G.M.d.C.)
3 Oswaldo Cruz Foundation, Aggeu Magalhães Research Center, Recife 50740-465, PE, Brazil; lindomarfiocruz@gmail.com
4 Institute of Biological and Health Sciences, Federal University of Alagoas, Maceió 57072-900, AL, Brazil; abelardo.iunior@icbs.ufal.br
5 Postgraduate Program in Natural and Synthetic Products Bioactive, Health Sciences Center, Federal University of Paraiba, João Pessoa 58051-900, PB, Brazil; yurimangueira@ltf.ufpb.br (Y.M.d.N.); jbarbosa@ltf.ufpb.br (J.M.B.-F.)
6 Graduate Program in Development and Technological Innovation in Medicines, Federal University of Campina Grande, Cajazeiras 58900-000, PB, Brazil
7 Department of Cell and Molecular Biology, Biotechnology Center, Federal University of Paraíba, João Pessoa 58051-900, PB, Brazil; kristerson@cbiotec.ufpb.br
* Correspondence: hdmcoutinho@gmail.com (H.D.M.C.); barbara@ltf.ufpb.br (B.V.d.O.S.)

Contents

1. Copies of IR, ^{1}H and ^{13}C NMR spectra of Caulerpin **(1)** **S2**
2. Copies of IR, ^{1}H and ^{13}C NMR spectra of product **2** **S3**
3. Copies of IR, ^{1}H NMR, ^{13}C NMR, HMBC spectra of product **3** **S5**
4. Copies of IR, ^{1}H and ^{13}C NMR spectra of product **4** **S7**
5. Copies of IR, ^{1}H NMR, ^{13}C NMR, COSY, HMQC and HMBC spectra of product **5** **S8**
6. Copies of IR, ^{1}H and ^{13}C NMR spectra of product **6** **S11**
7. Copies of ^{1}H NMR, ^{13}C NMR and HMBC spectra of product **7** **S13**
8. Copies of ^{1}H NMR, ^{13}C NMR, COSY, HMQC and HMBC spectra of product **8** **S14**
9. Copies of IR, ^{1}H and ^{13}C NMR spectra of product **9** **S17**
10. Copies of IR, ^{1}H and ^{13}C NMR spectra of product **10** **S18**
11. Copies of IR, ^{1}H NMR, ^{13}C NMR and HMBC spectra of product **11** **S20**
12. Copies of IR, ^{1}H and ^{13}C NMR spectra of product **12** **S22**
13. Copies of IR, ^{1}H NMR, ^{13}C NMR and HMBC spectra of product **13** **S23**
14. Copies of ^{1}H and ^{13}C NMR spectra of product **14** **S25**
15. Copies of ^{1}H and ^{13}C NMR spectra of product **15** **S26**

1. Copies of IR, ^{1}H and ^{13}C NMR spectra of Caulerpin (**1**)

Figure S1. IR of caulerpin (**1**)

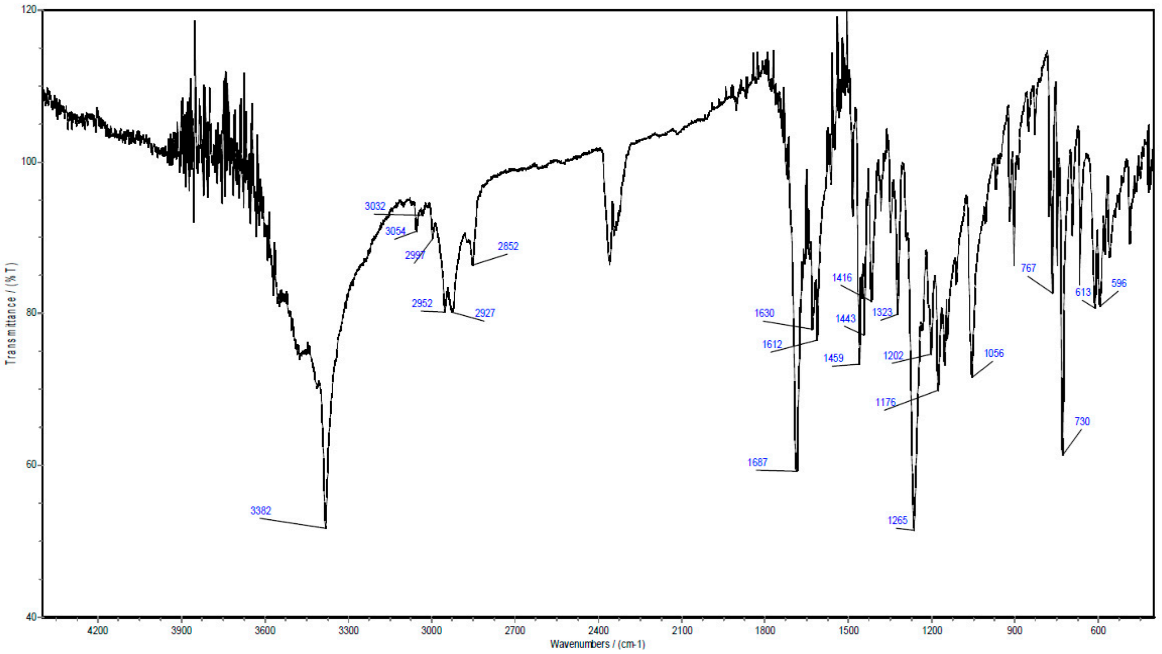

Figure S2. ^{1}H NMR of **1** (200 MHz, $CDCl_3$).

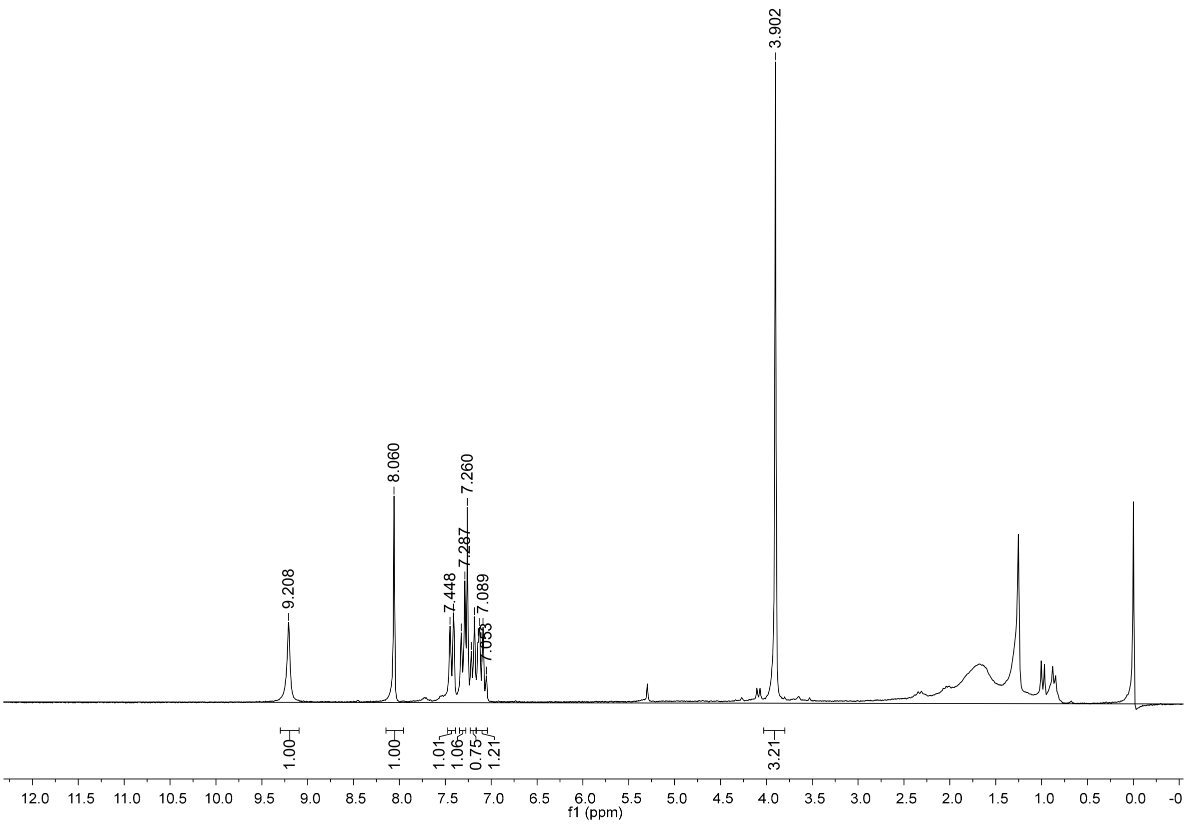

Figure S3. ^{13}C NMR of **1** (200 MHz, $CDCl_3$).

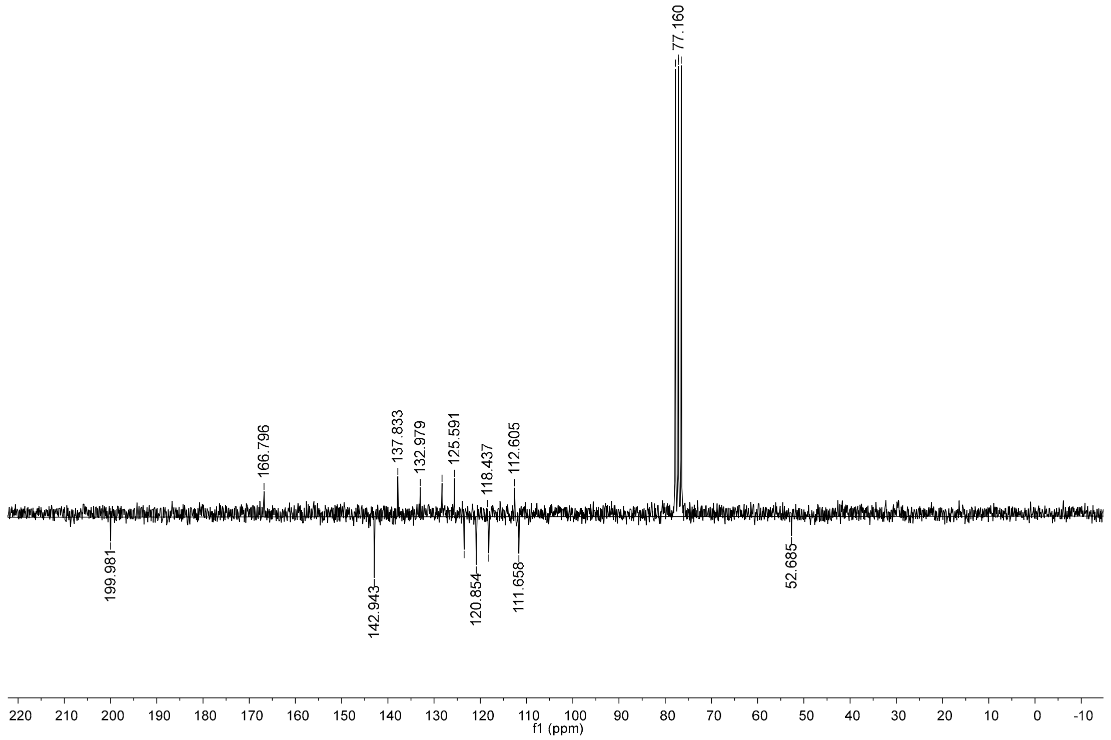

2. Copies of IR, ^{1}H and ^{13}C NMR spectra of product **2**

Figure S4. IR of **2**

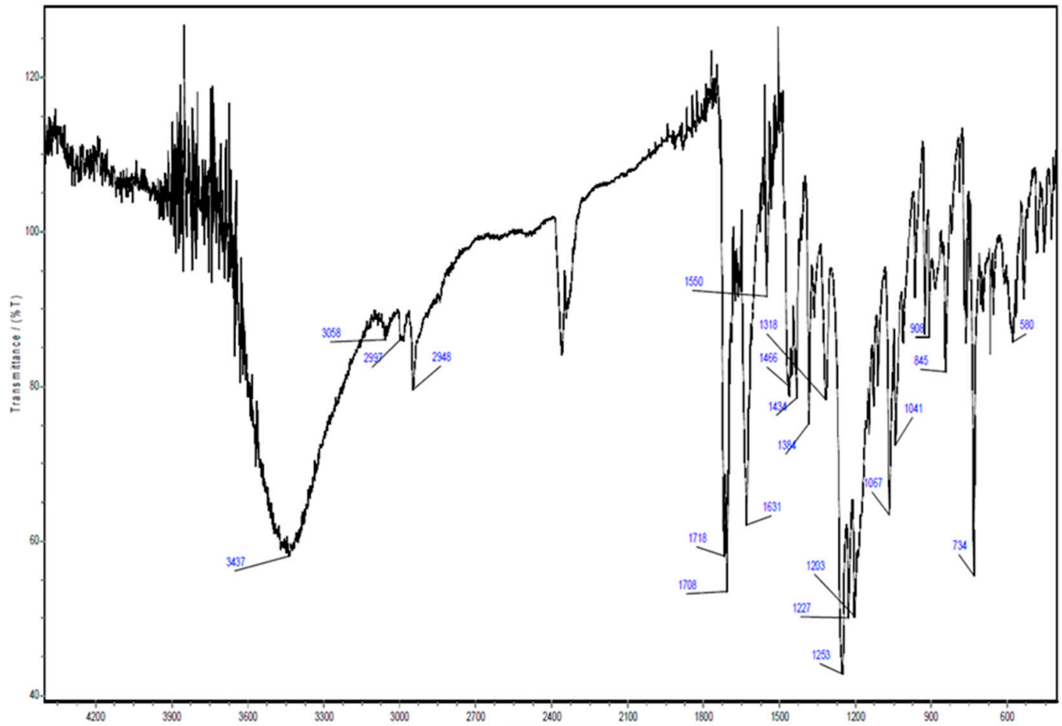

Figure S5. ^{1}H NMR of **2** (200 MHz, $CDCl_3$).

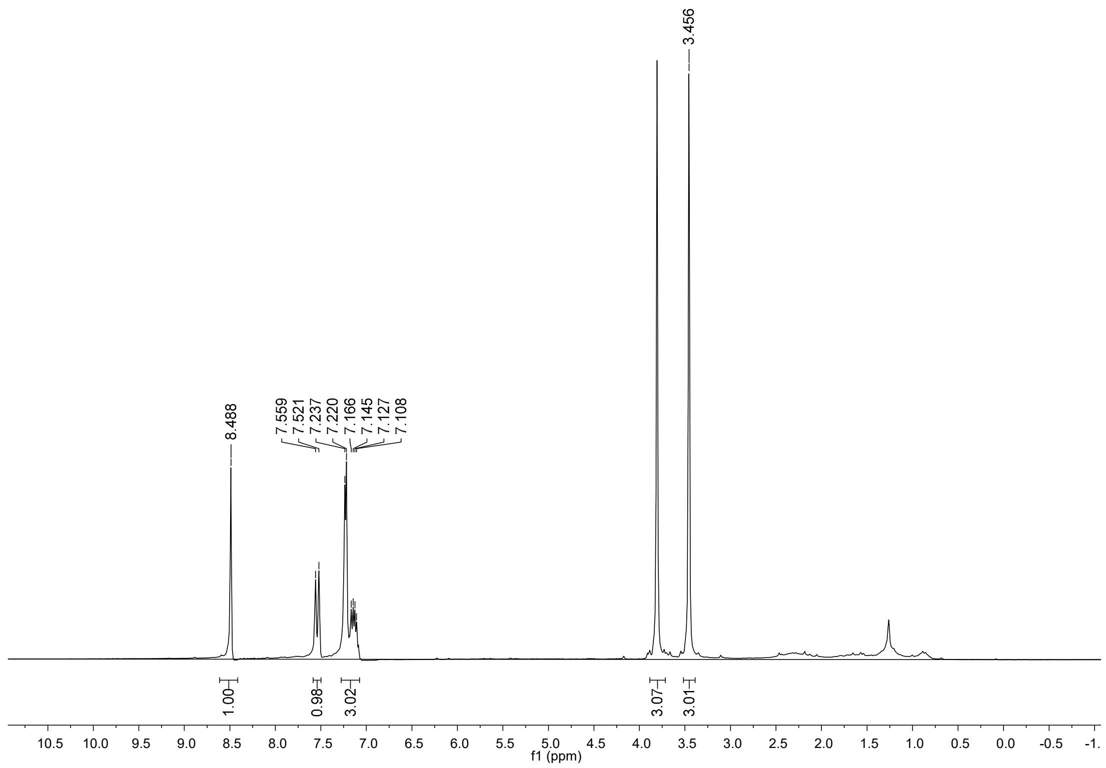

Figure S6. ^{13}C NMR of **2** (200 MHz, $CDCl_3$).

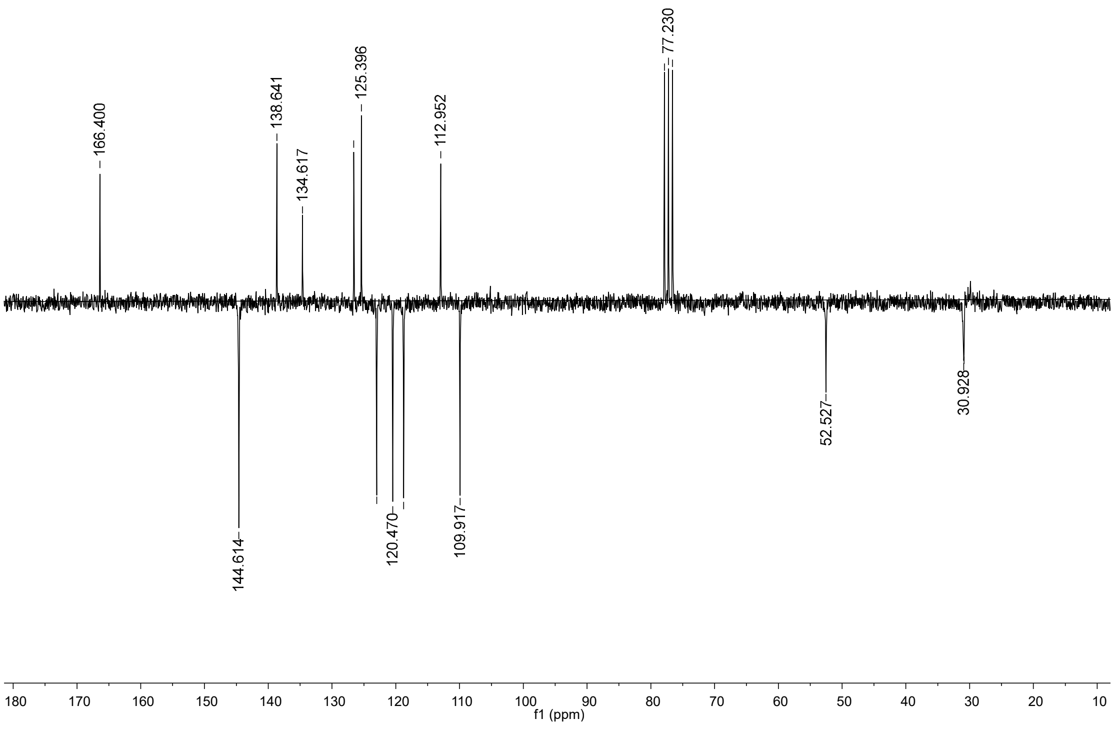

3. Copies of IR, ^{1}H NMR, ^{13}C NMR, HMBC spectra of product **3**

Figure S7. IR of **3**

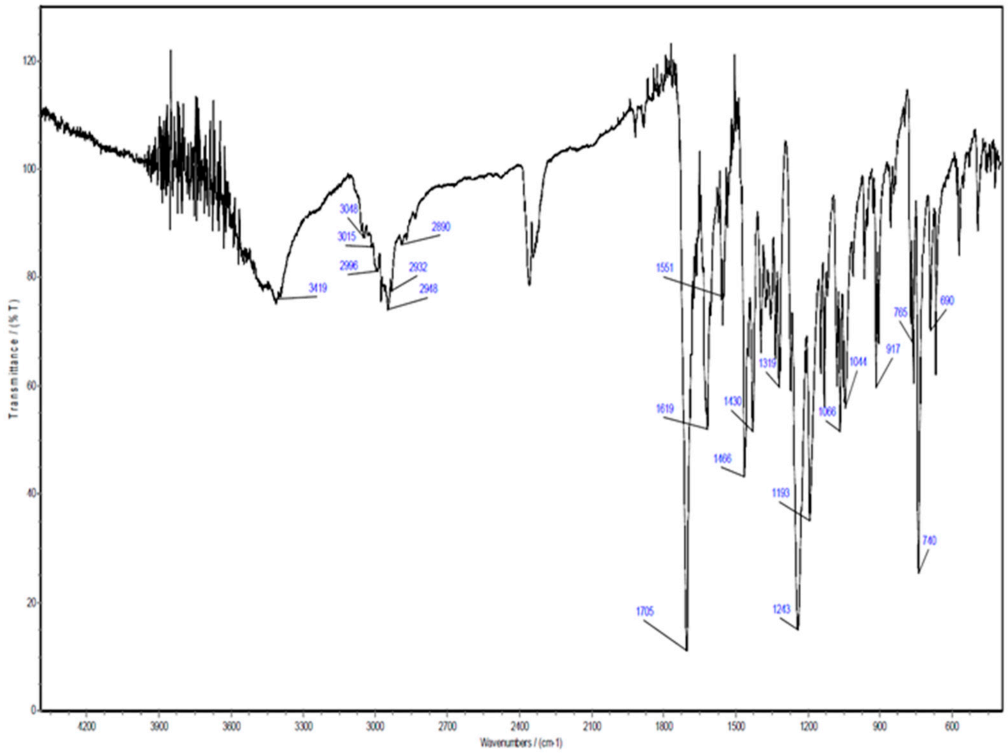

Figure S8. ^{1}H NMR of **3** (200 MHz, $CDCl_3$).

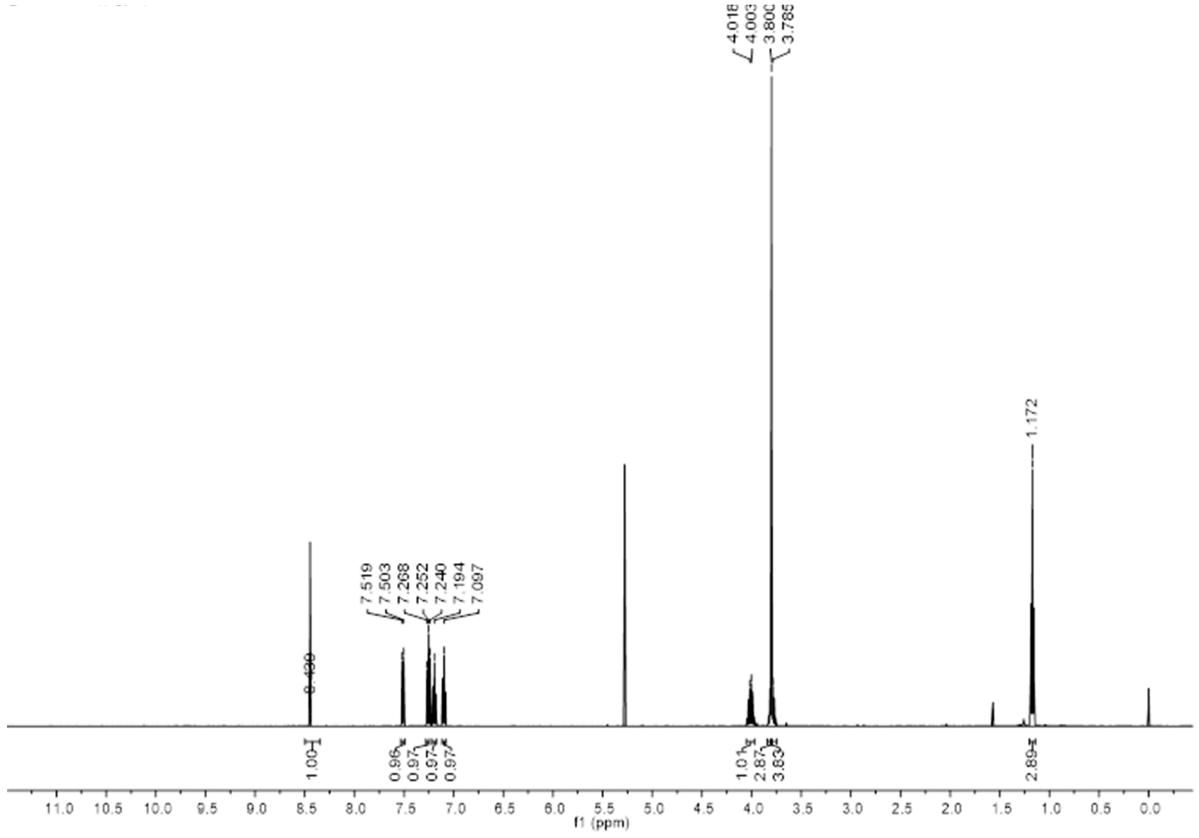

Figure S9. ^{13}C NMR of **3** (500 MHz, $CDCl_3$).

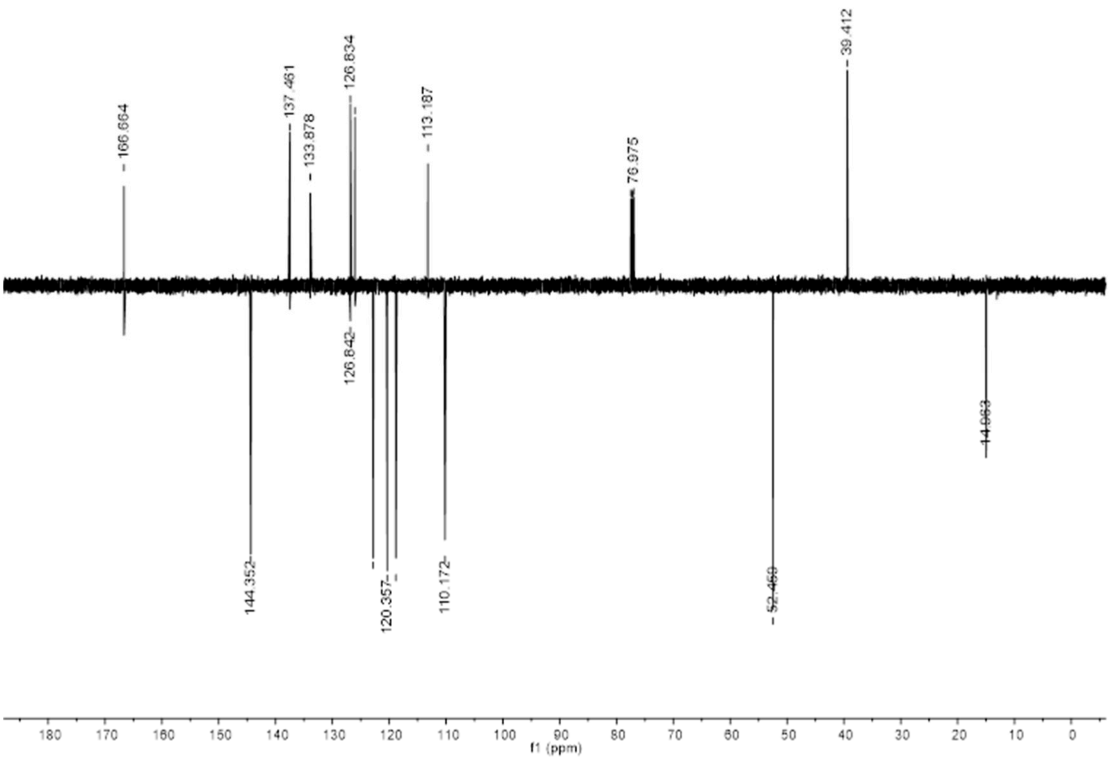

Figure S10. HMBC of **3** (500 MHz, $CDCl_3$).

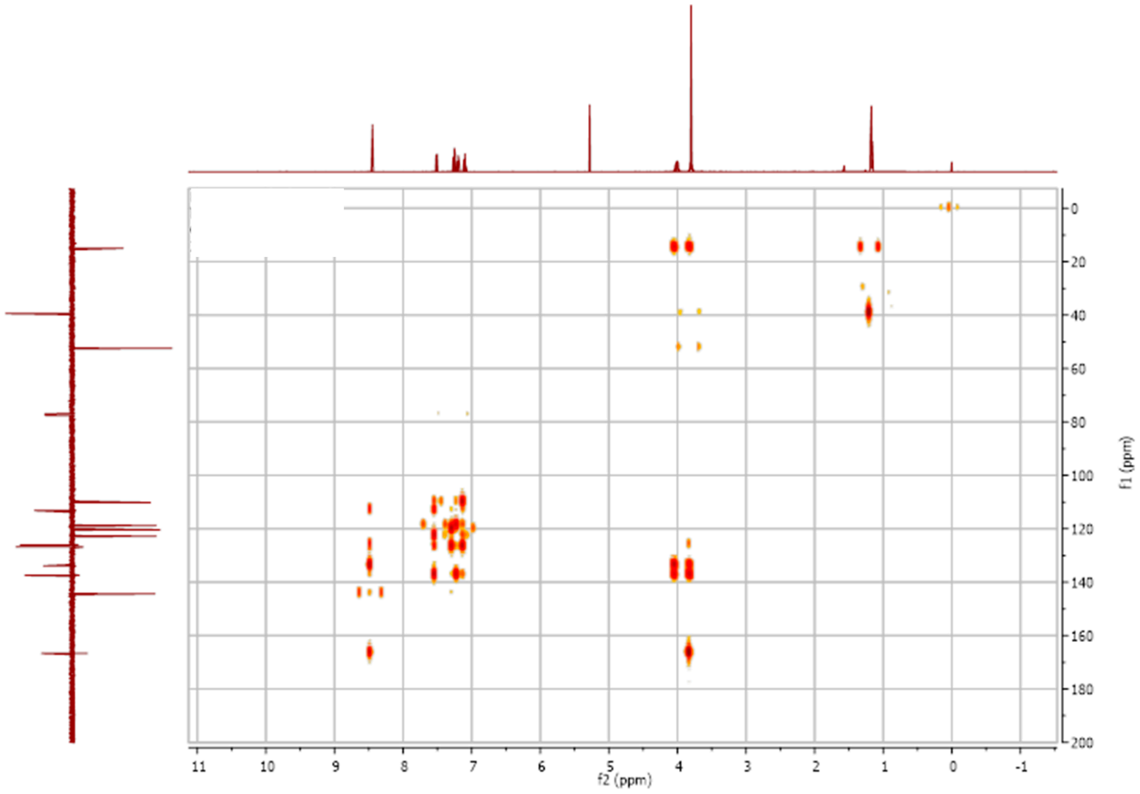

4. Copies of IR, ^{1}H and ^{13}C NMR spectra of product **4**

Figure S11. IR of **4**

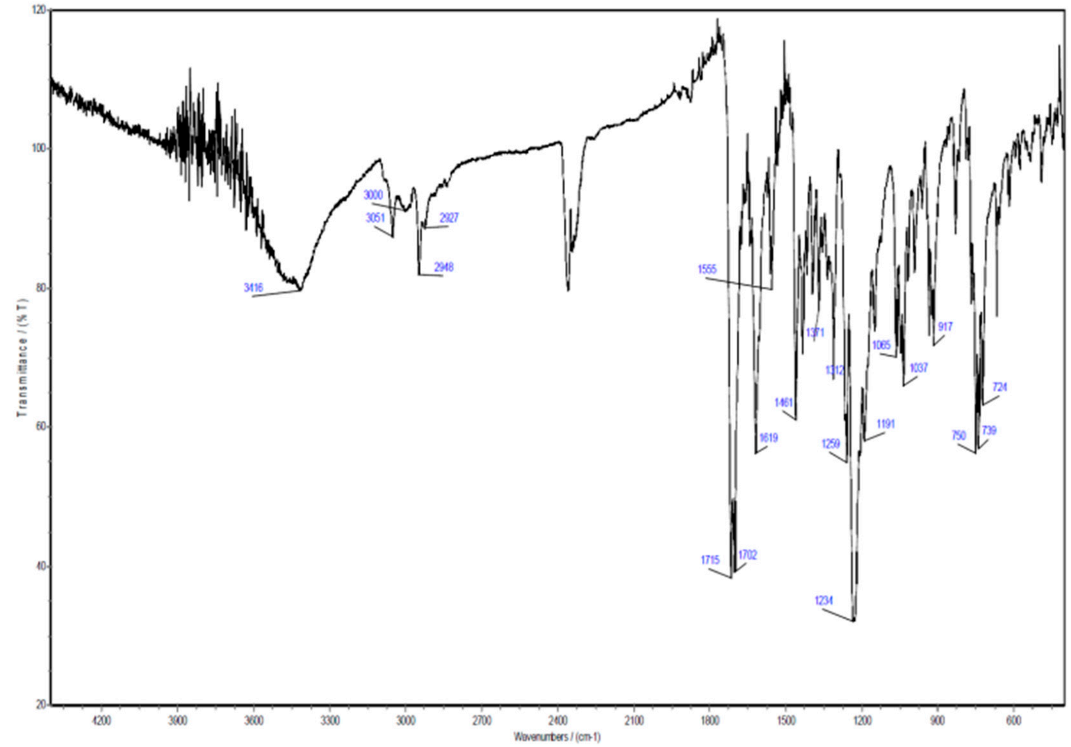

Figure S12. ^{1}H NMR of **4** (200 MHz, $CDCl_3$).

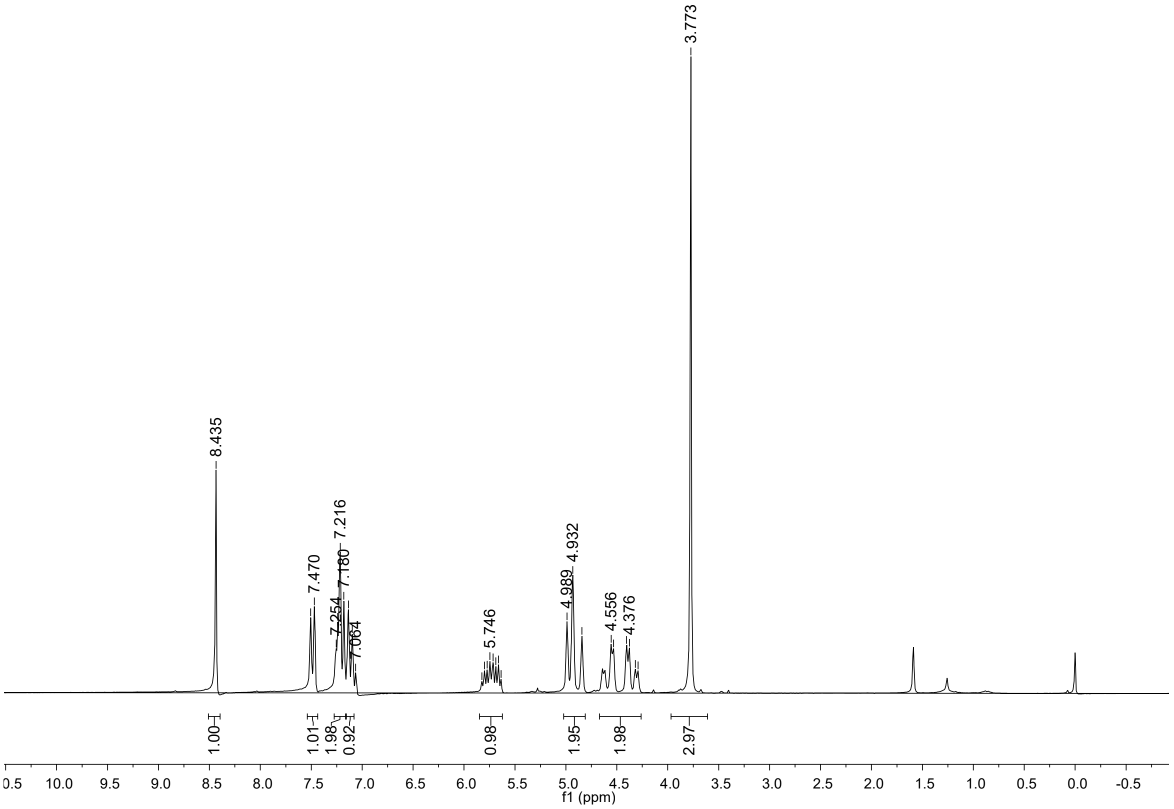

Figure S13. ^{13}C NMR of **4** (200 MHz, $CDCl_3$).

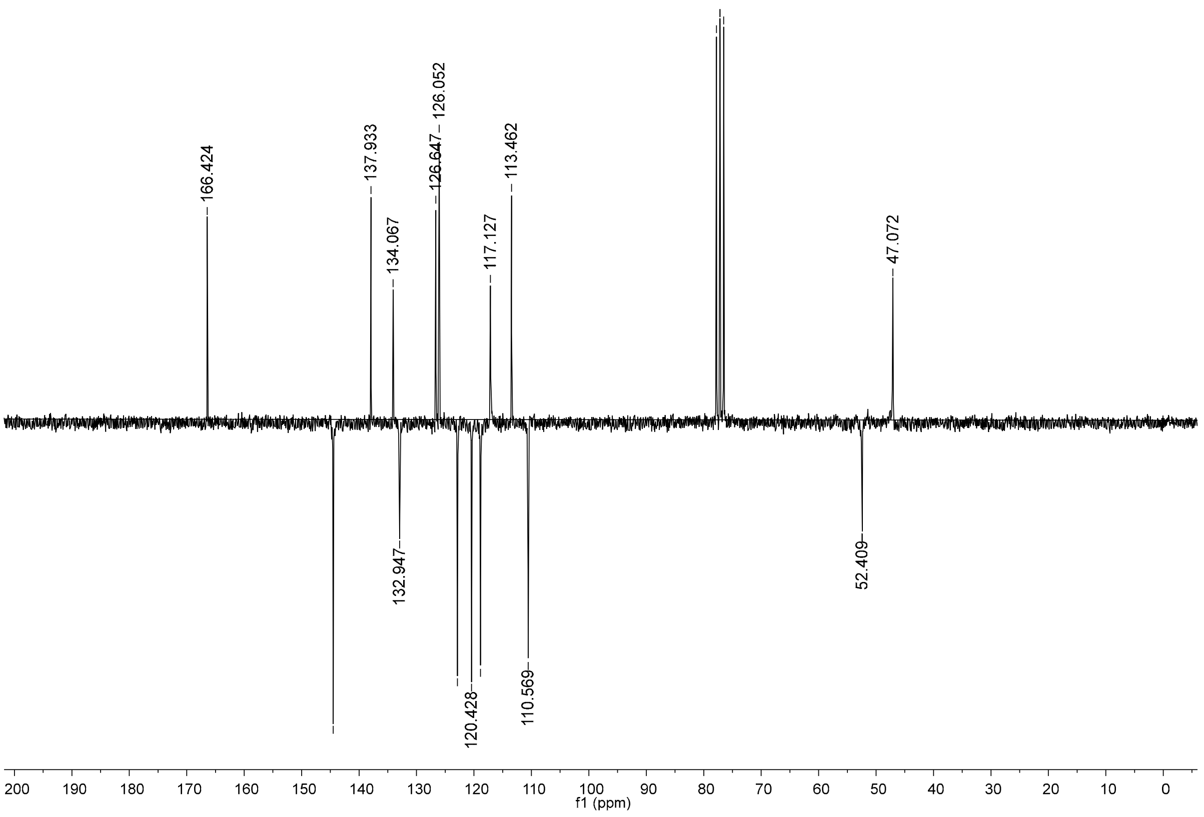

5. Copies of IR, ^{1}H NMR, ^{13}C NMR, COSY, HMQC and HMBC spectra of product **5**

Figure S14. IR of **5**

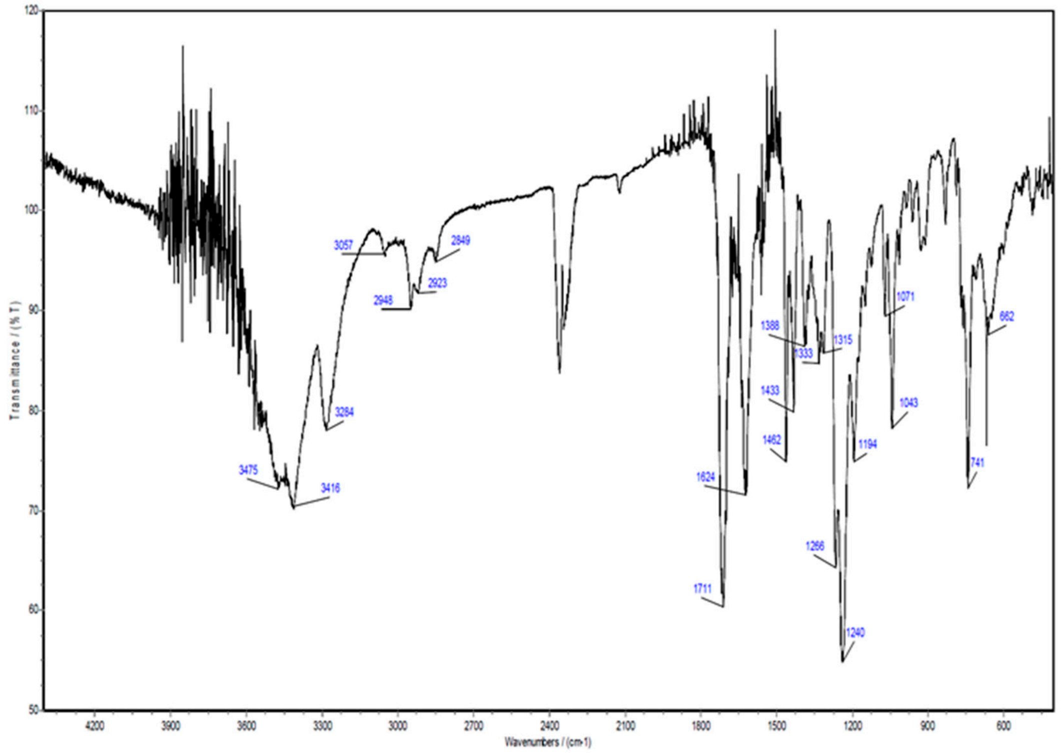

Figure S15. ^{1}H NMR of **5** (500 MHz, $CDCl_3$).

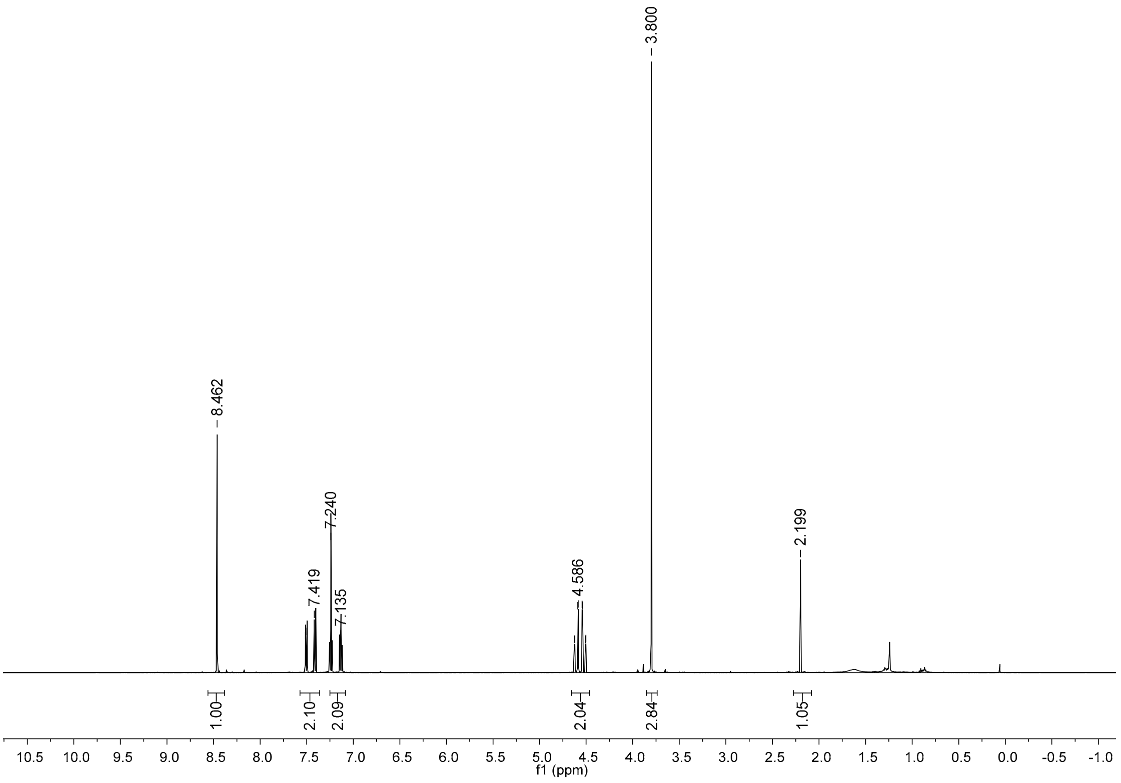

Figure S16. ^{13}C NMR of **5** (500 MHz, $CDCl_3$).

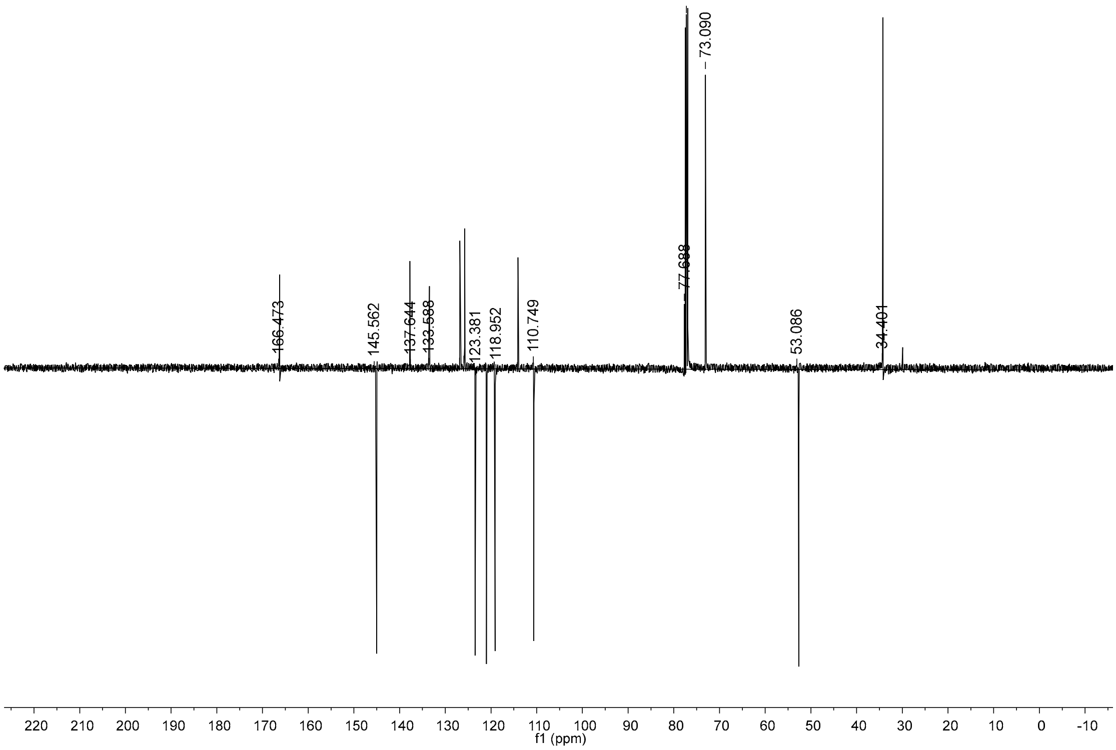

Figure S17. COSY of **5** (500 MHz, $CDCl_3$).

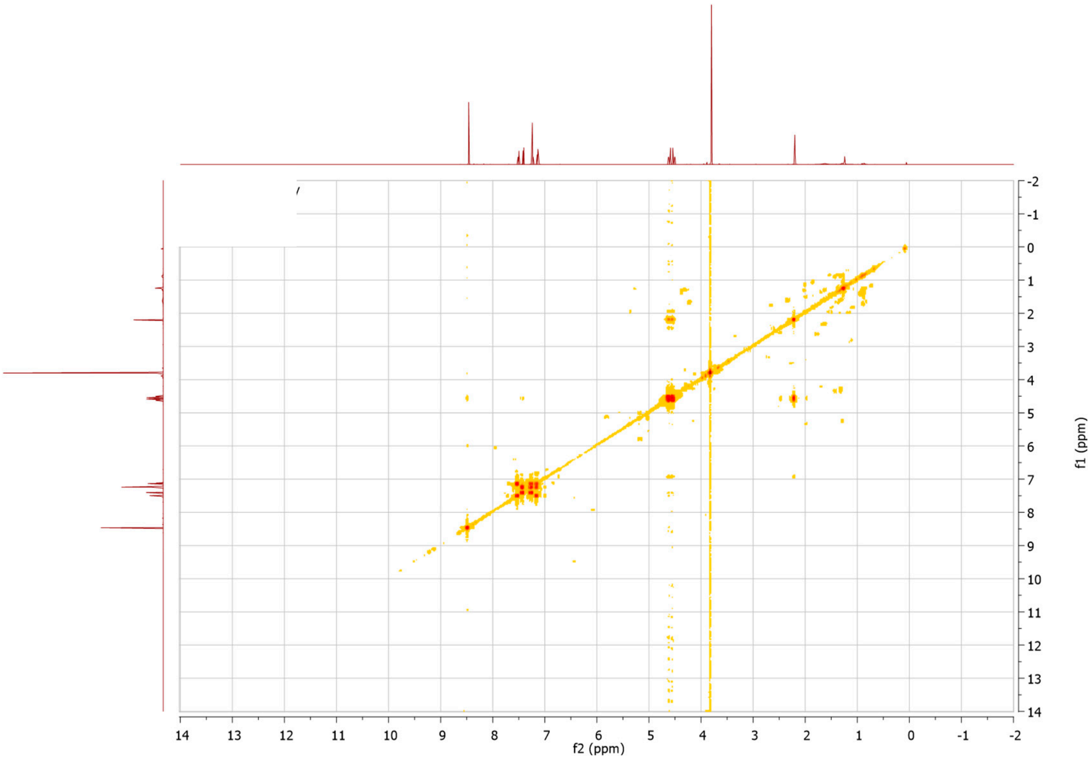

Figure S18. HMQC of **5** (500 MHz, $CDCl_3$).

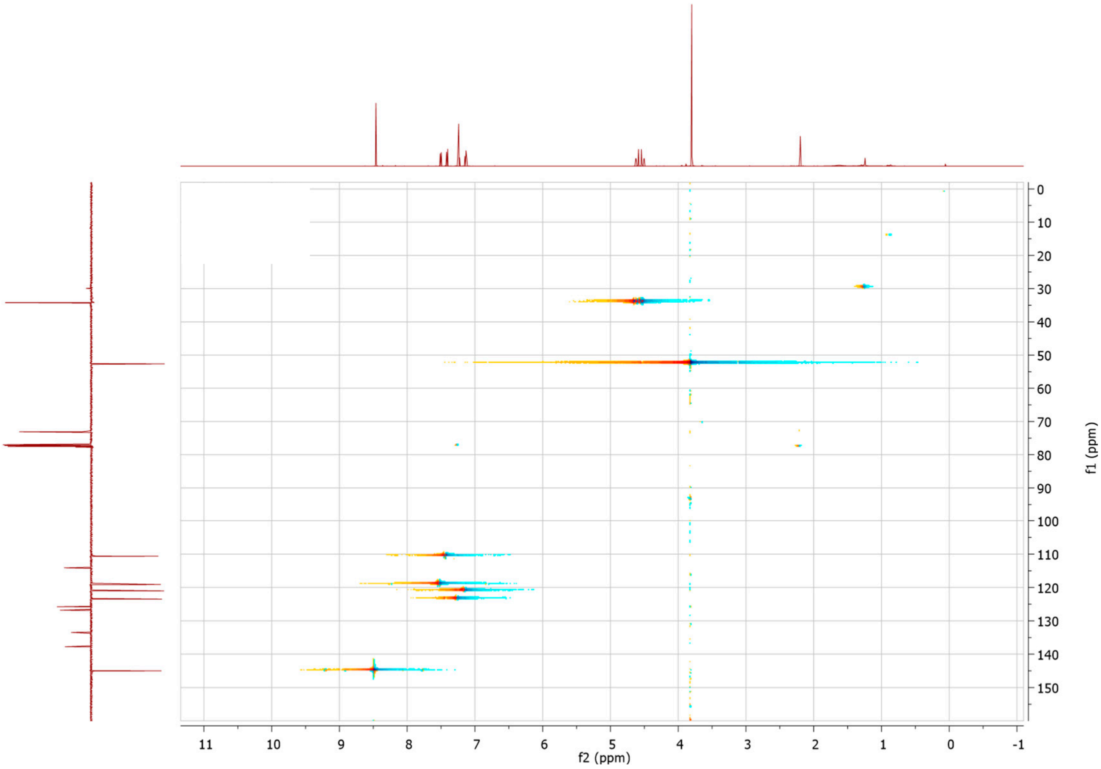

Figure S19. HMBC of **5** (500 MHz, $CDCl_3$).

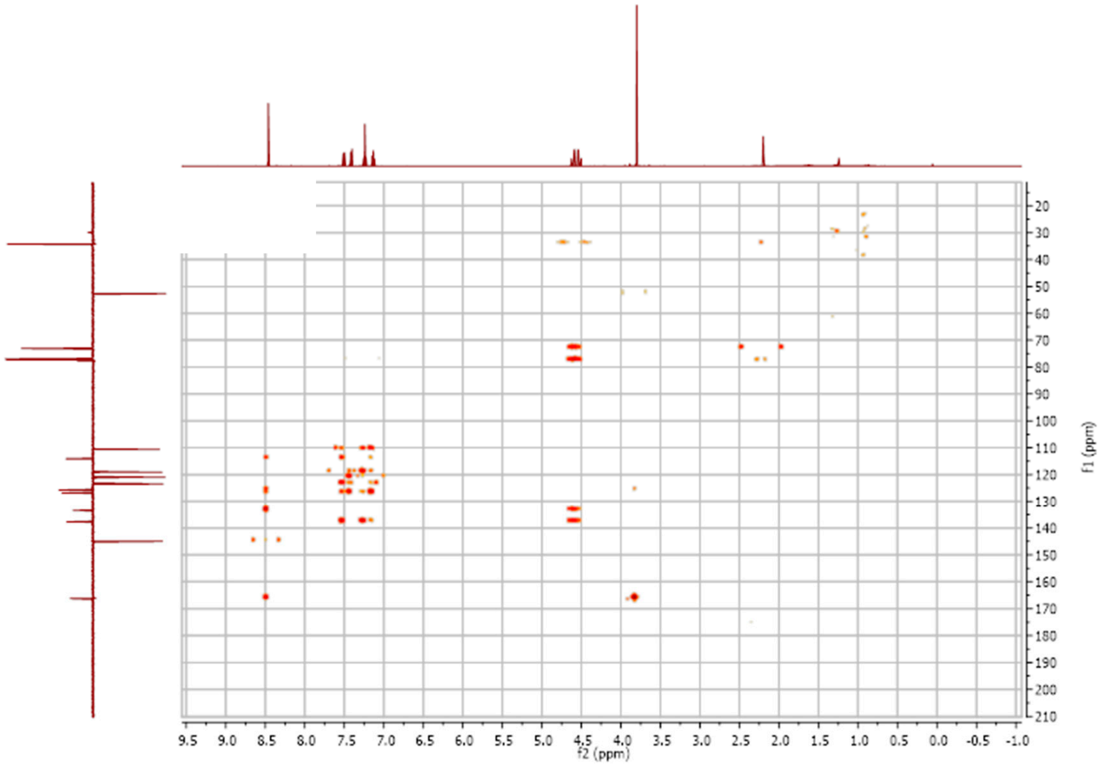

6. Copies of IR, ^{1}H and ^{13}C NMR spectra of product **6**

Figure S20. IR of **6**

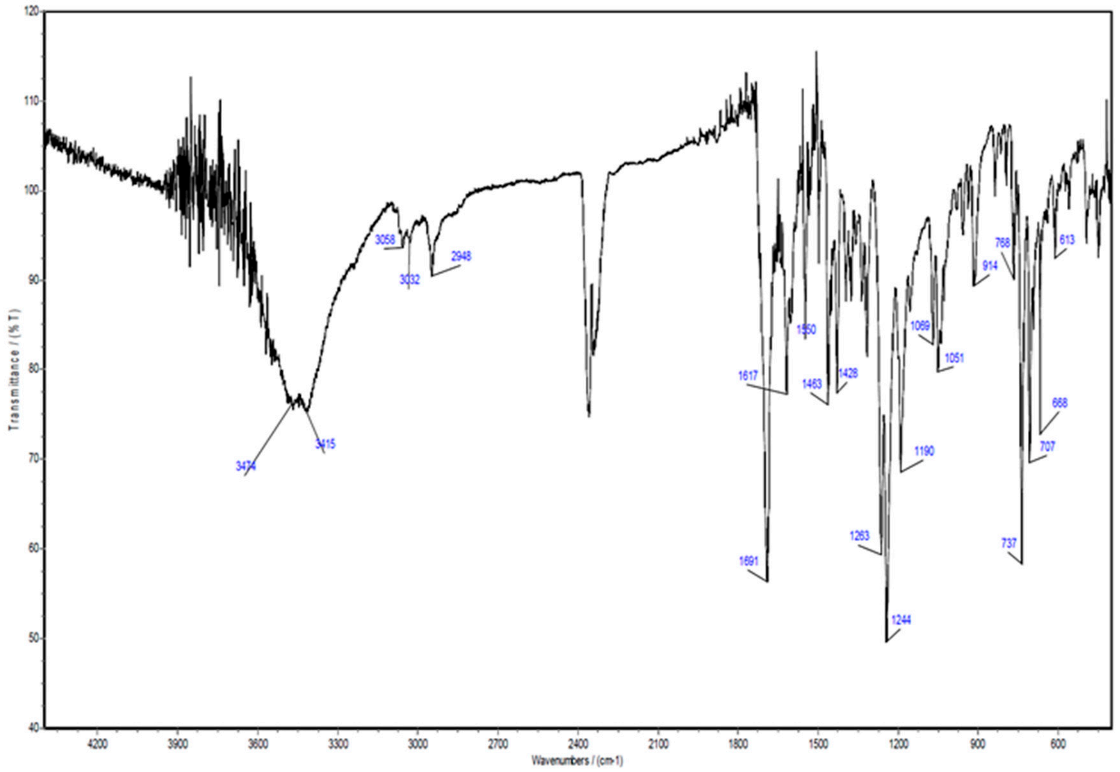

Figure S21. ^{1}H NMR of **6** (200 MHz, $CDCl_3$).

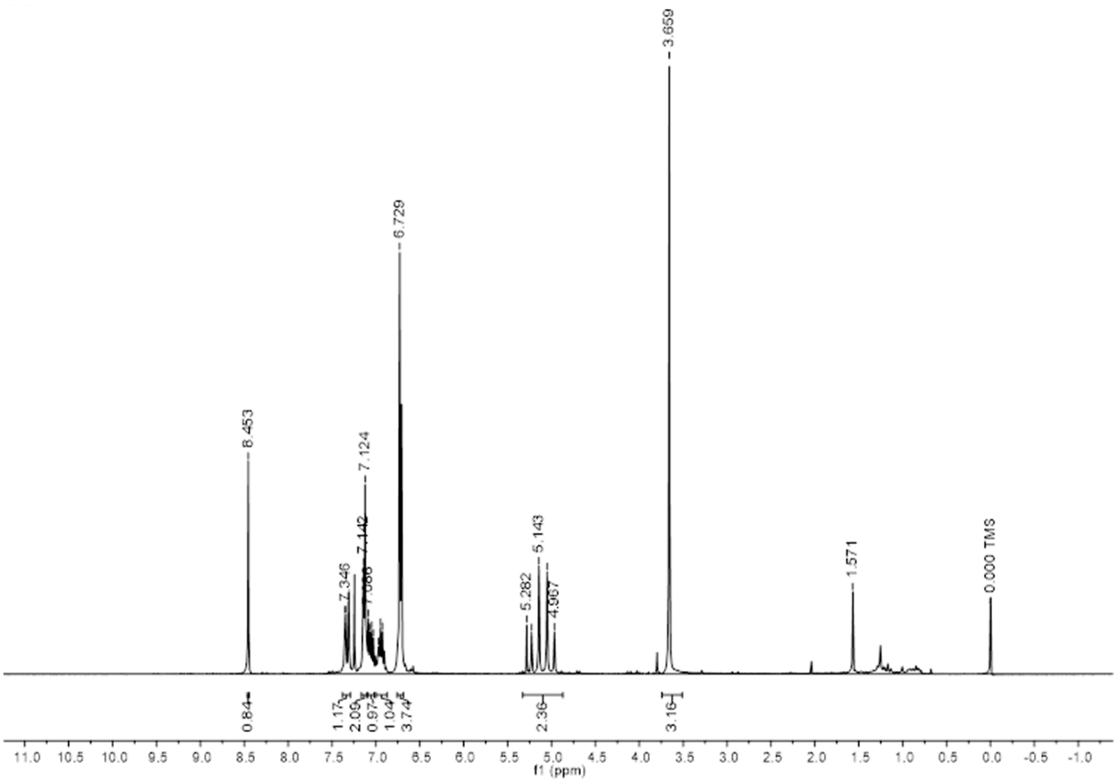

Figure S22. ^{13}C NMR of **6** (200 MHz, $CDCl_3$).

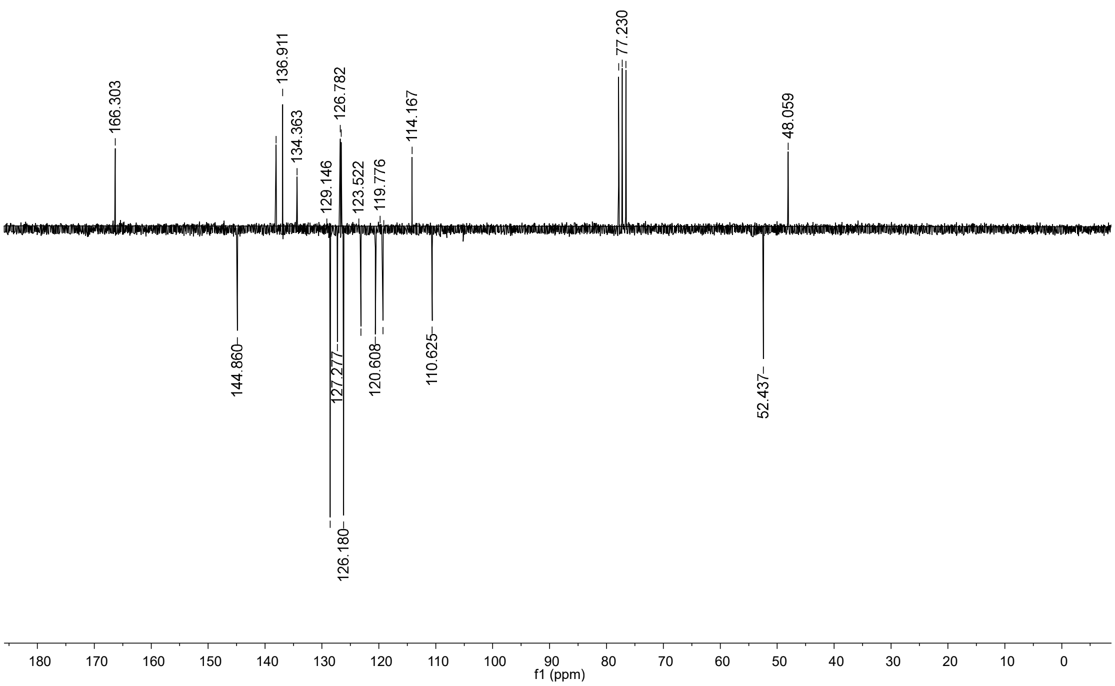

7. Copies of ^{1}H NMR, ^{13}C NMR and HMBC spectra of product **7**

Figure S23. ^{1}H NMR of **7** (500 MHz, MeOD).

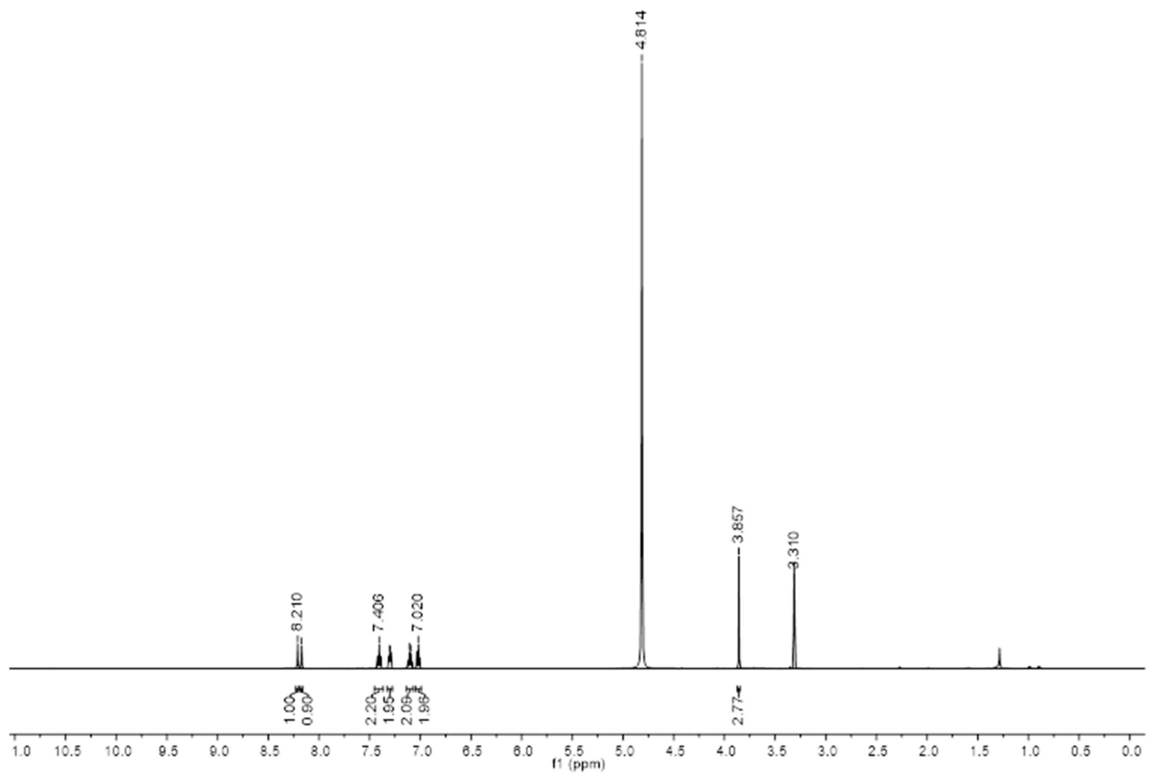

Figure S24. ^{13}C NMR of **7** (500 MHz, MeOD).

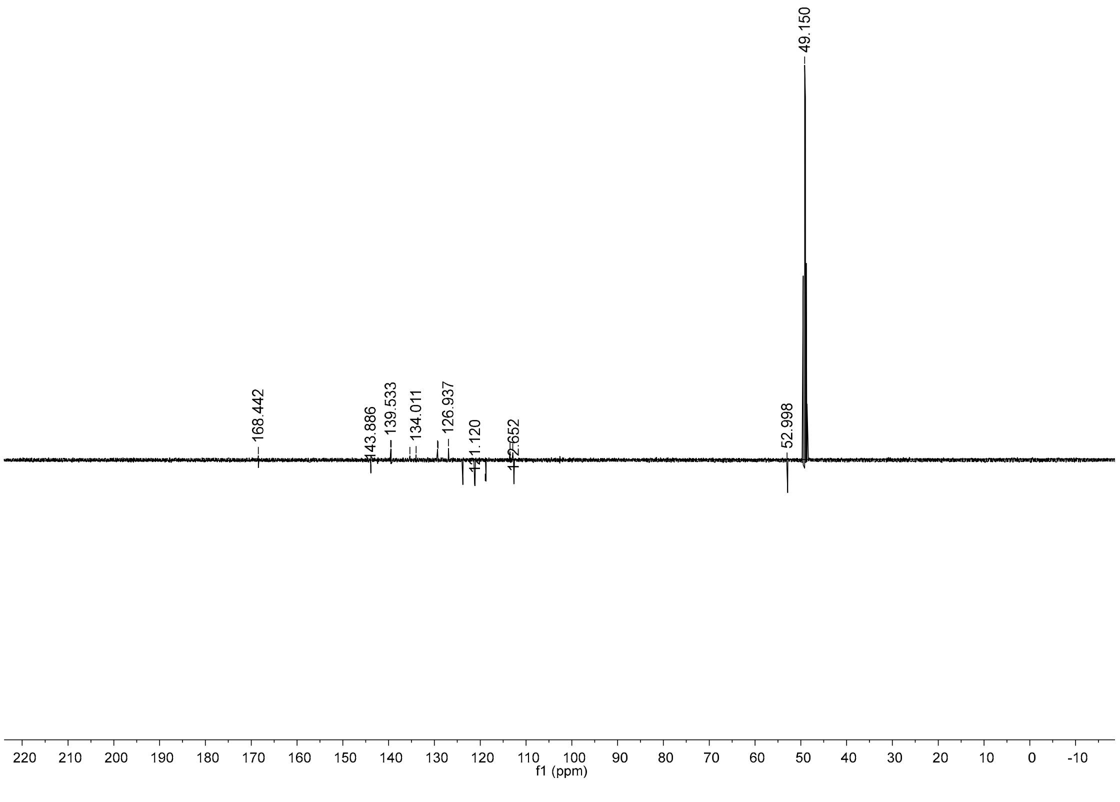

Figure S25. HMBC of **7** (500 MHz, MeOD).

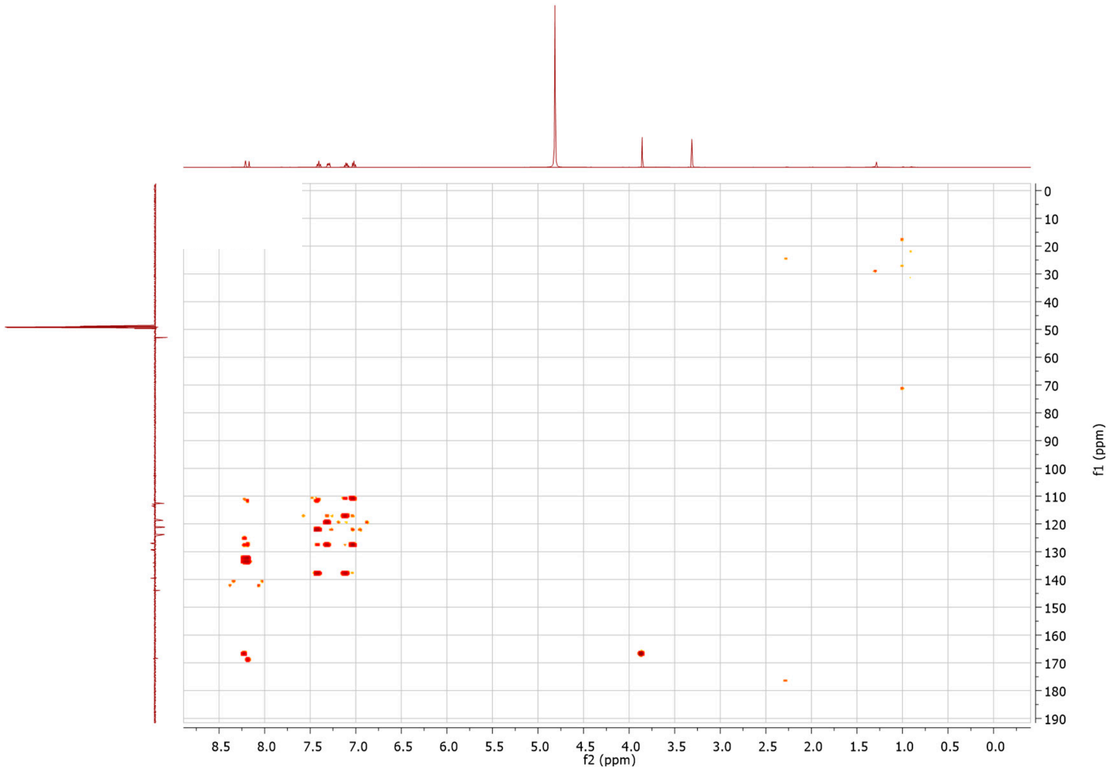

8. Copies of ^{1}H NMR, ^{13}C NMR, COSY, HMQC and HMBC spectra of product **8**

Figure S26. ^{1}H NMR of **8** (500 MHz, $CDCl_3$).

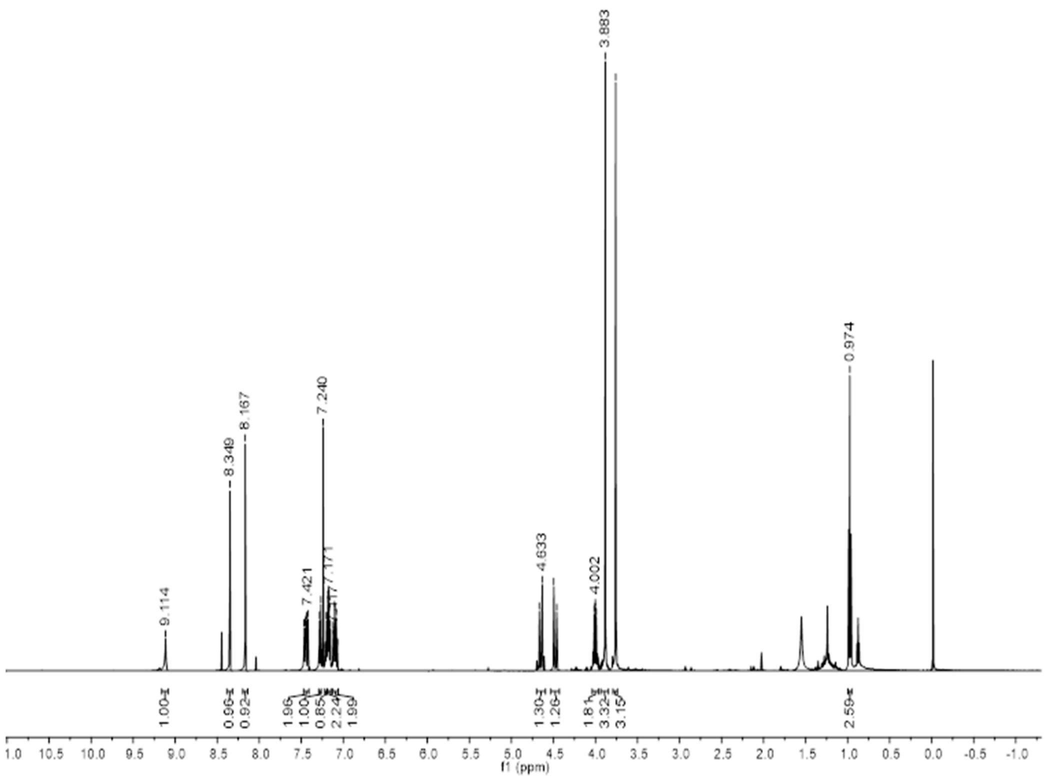

Figure S27. ^{13}C NMR of **8** (500 MHz, $CDCl_3$).

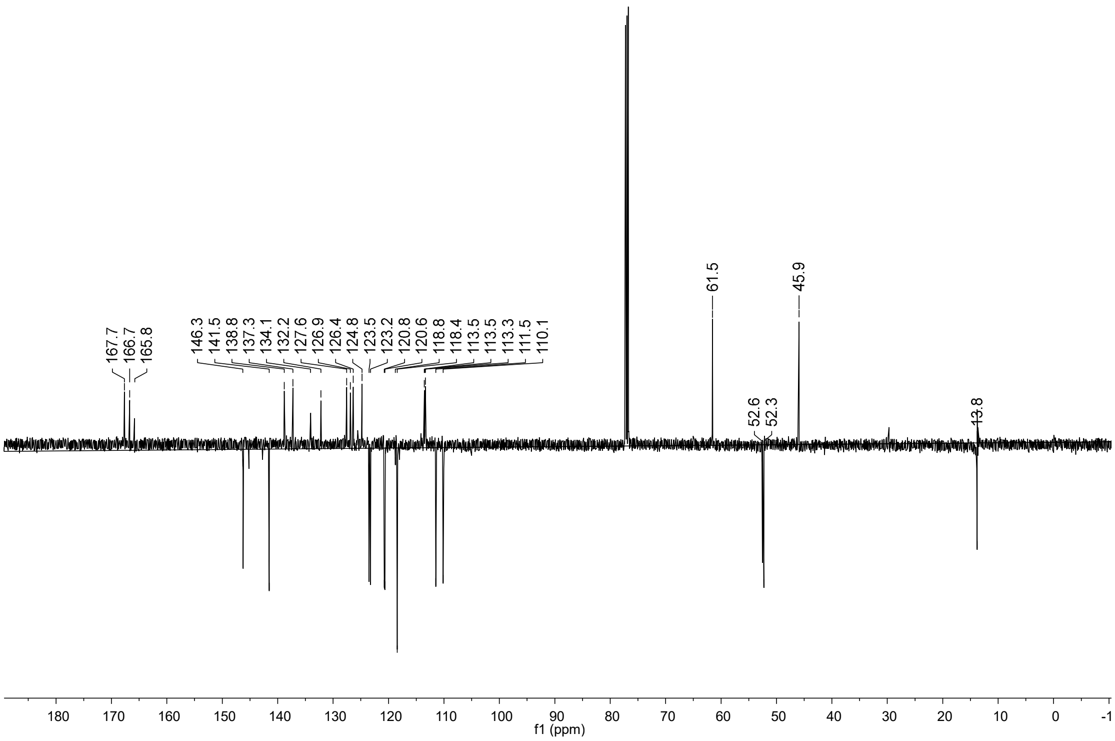

Figure S28. COSY of **8** (500 MHz, $CDCl_3$).

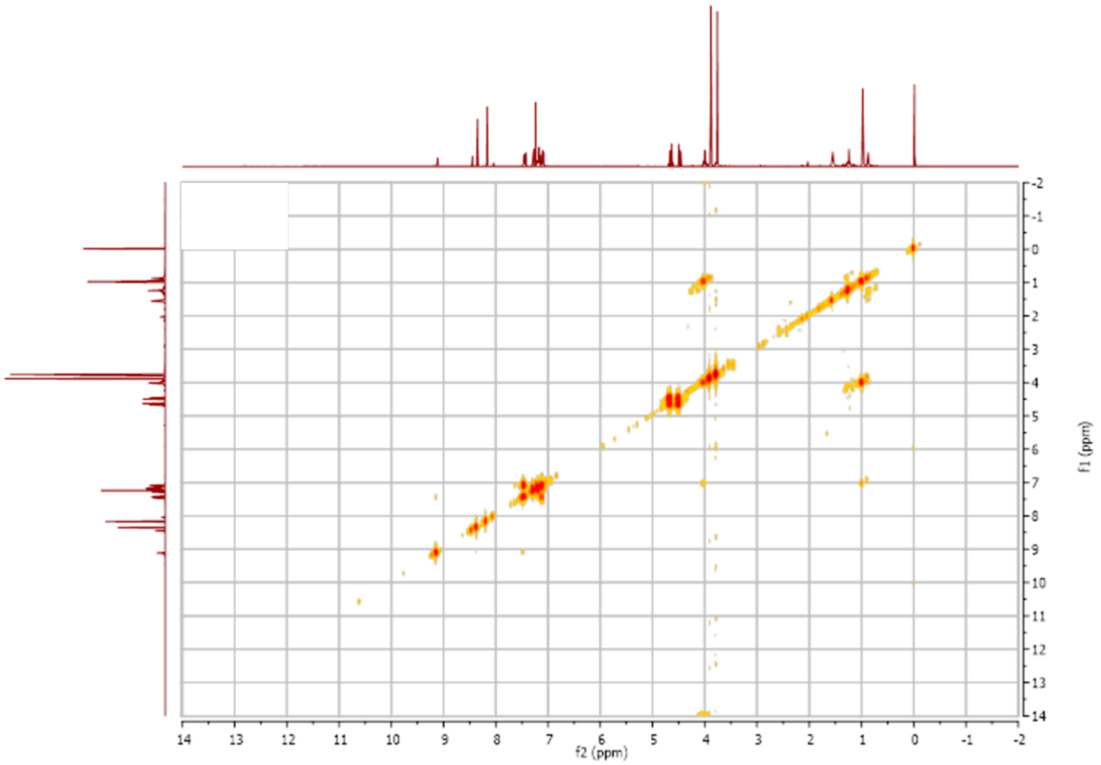

Figure S29. HMQC of **8** (500 MHz, $CDCl_3$).

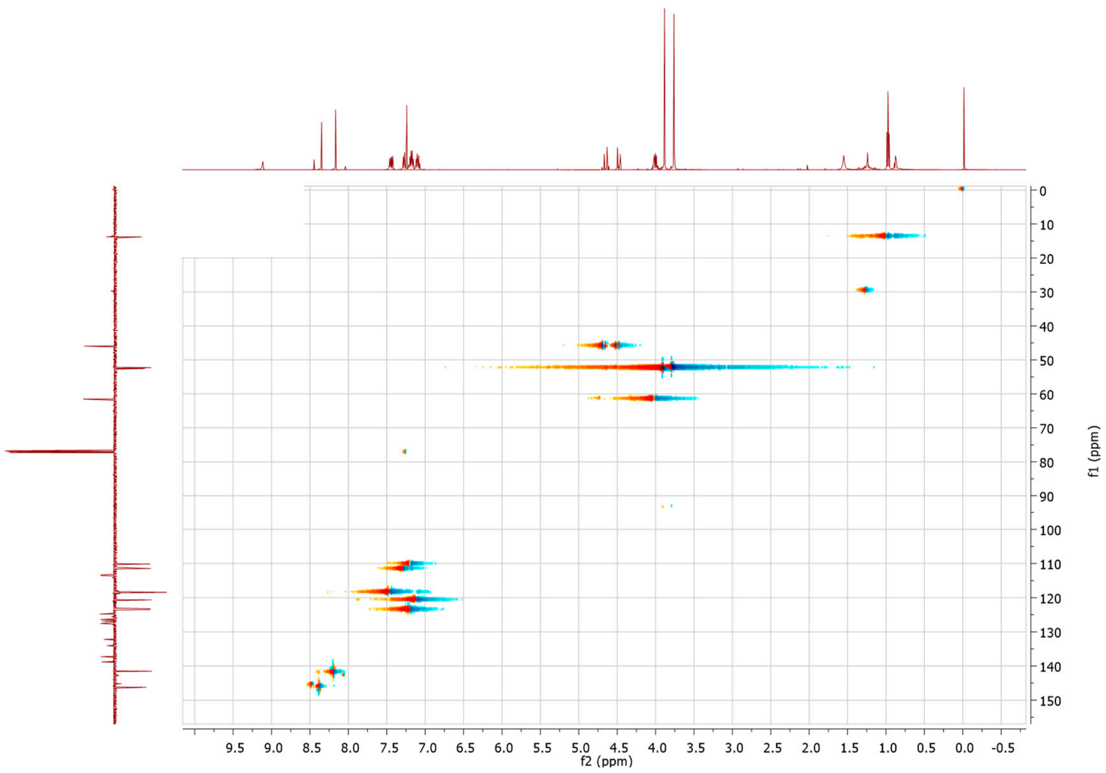

Figure S30. HMBC of **8** (500 MHz, $CDCl_3$).

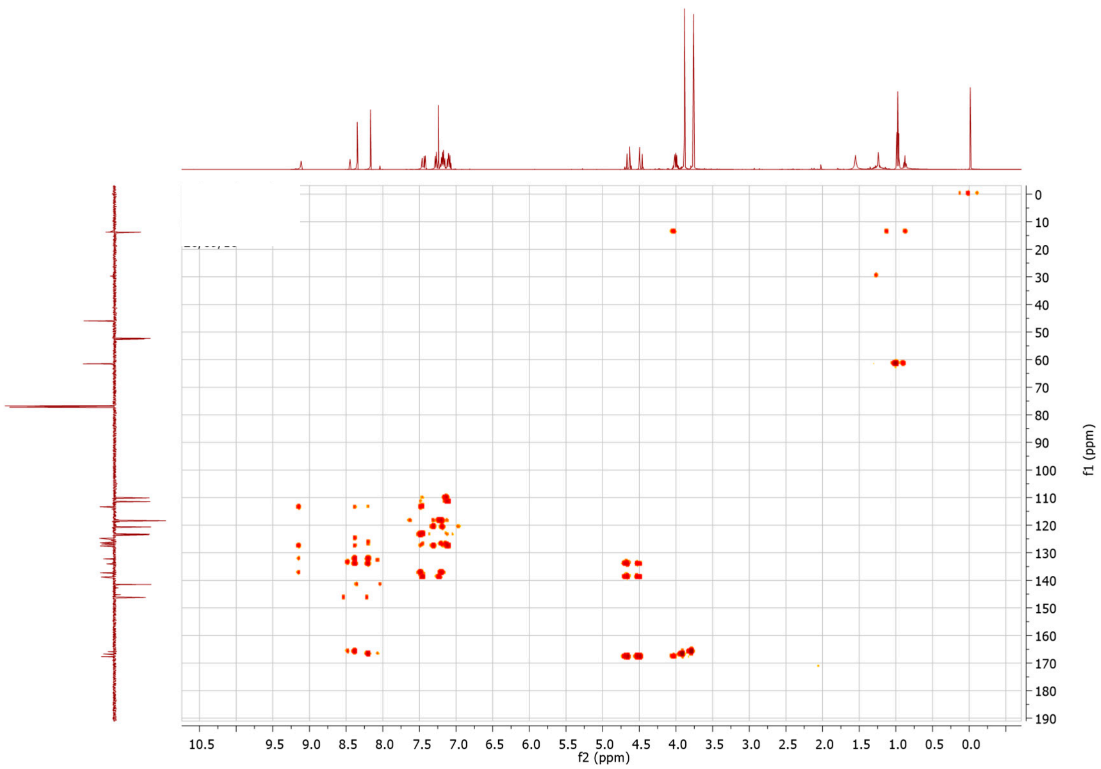

9. Copies of IR, ^{1}H and ^{13}C NMR spectra of product **9**

Figure S31. IR of **9**

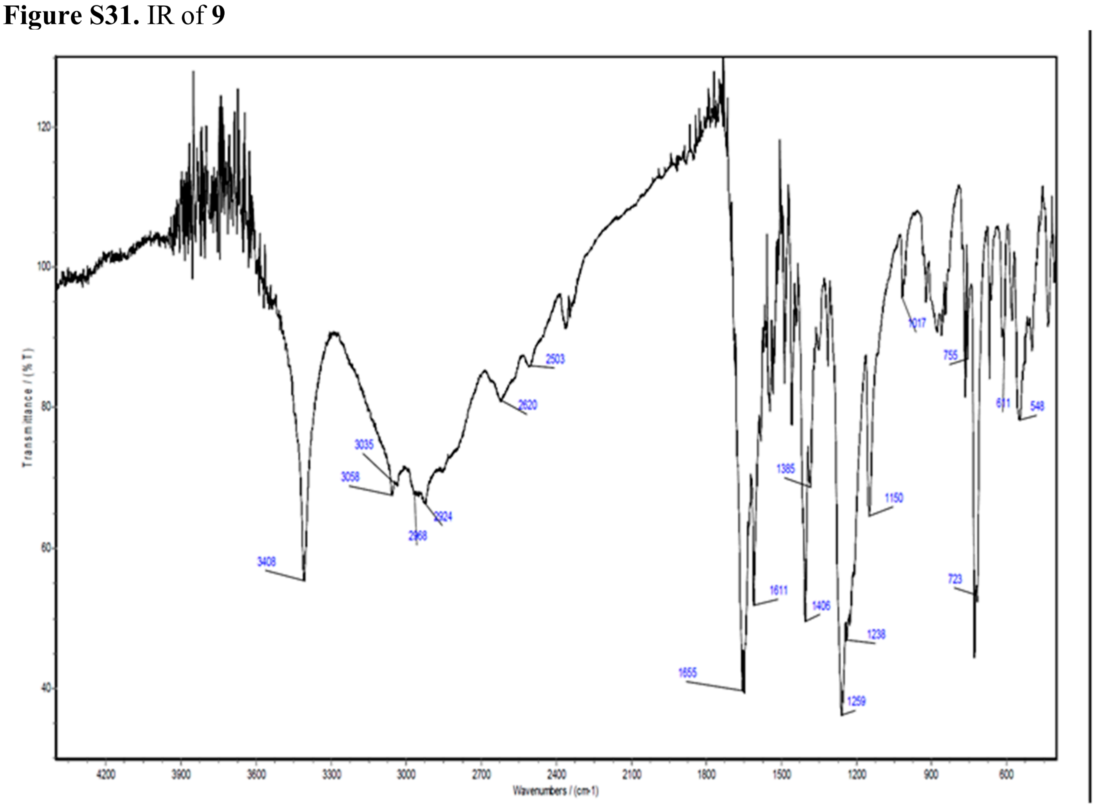

Figure S32. ^{1}H NMR of **9** (200 MHz, MeOD).

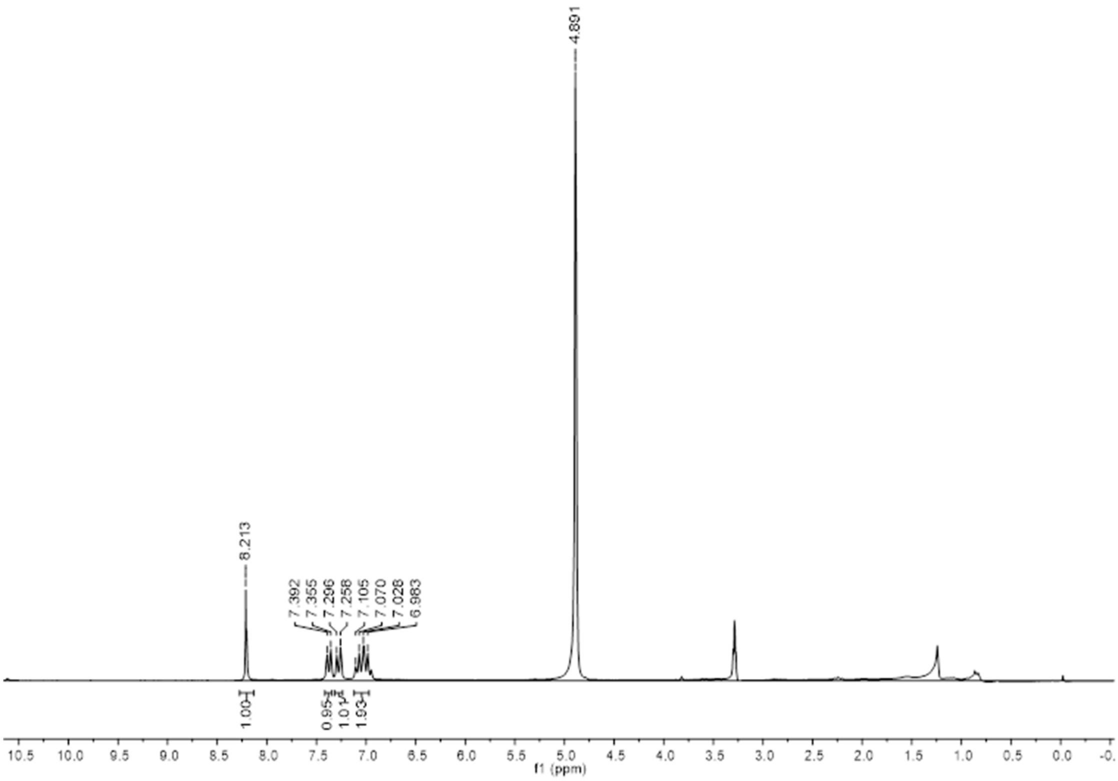

Figure S33. ^{13}C NMR of **9** (200 MHz, MeOD).

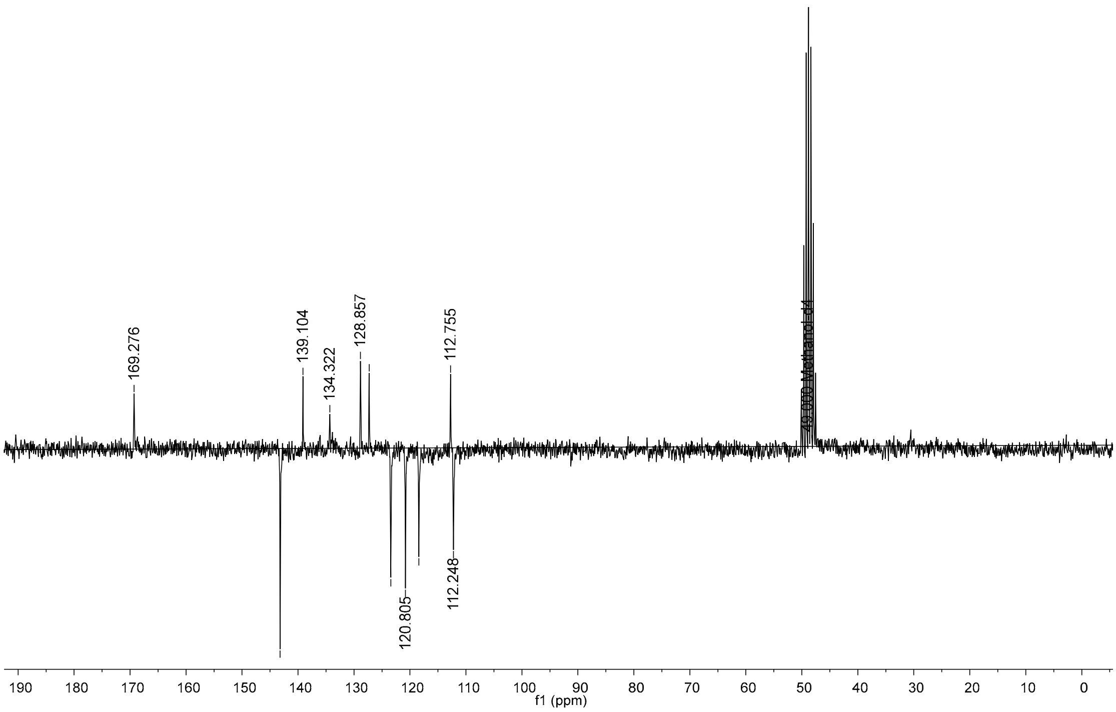

10. Copies of IR, ^{1}H and ^{13}C NMR spectra of product **10**

Figure S34. IR of **10**

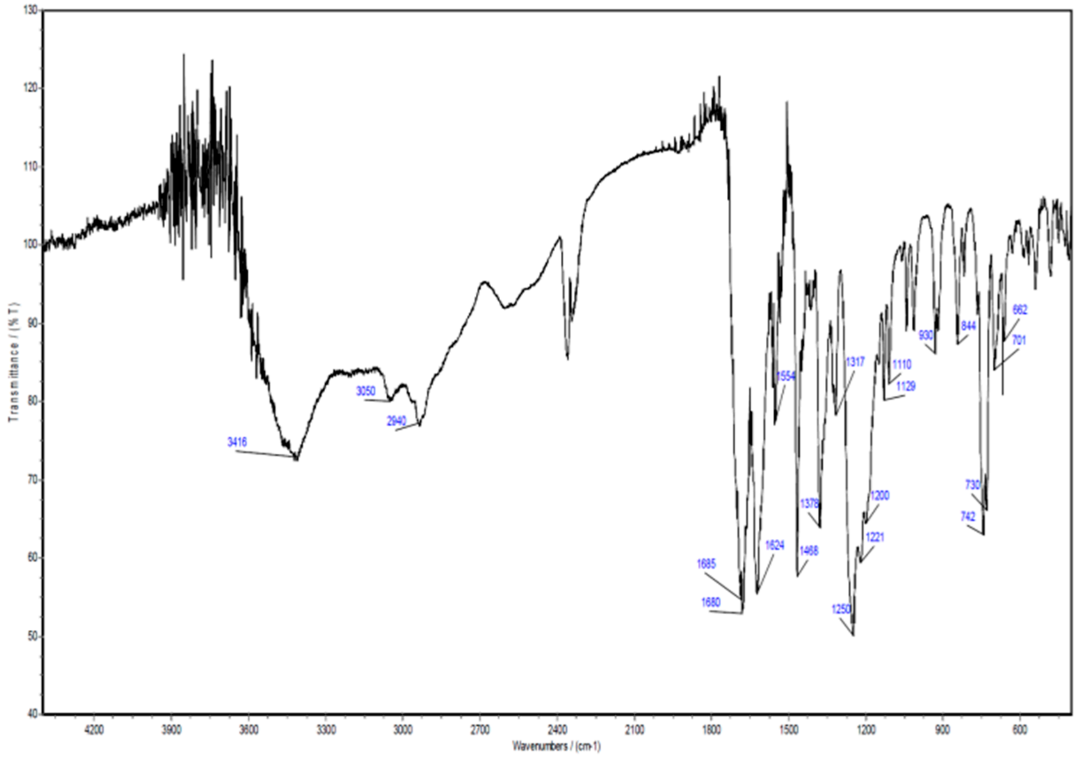

Figure S35. ^{1}H NMR of **10** (200 MHz, MeOD).

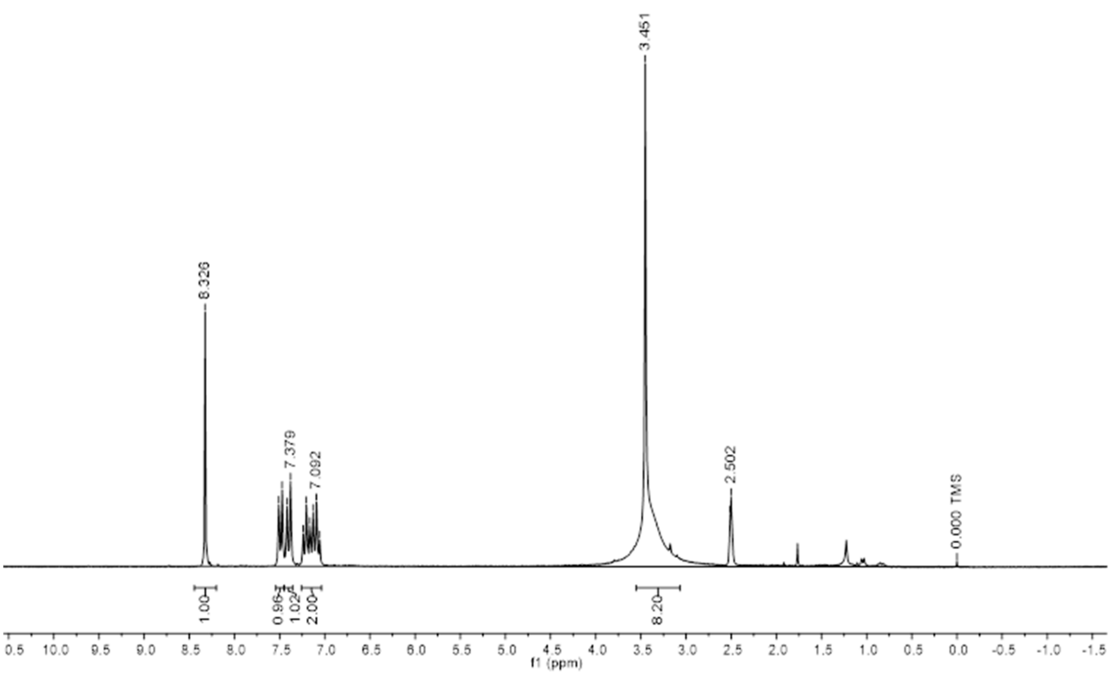

Figure S36. ^{13}C NMR of **10** (200 MHz, MeOD).

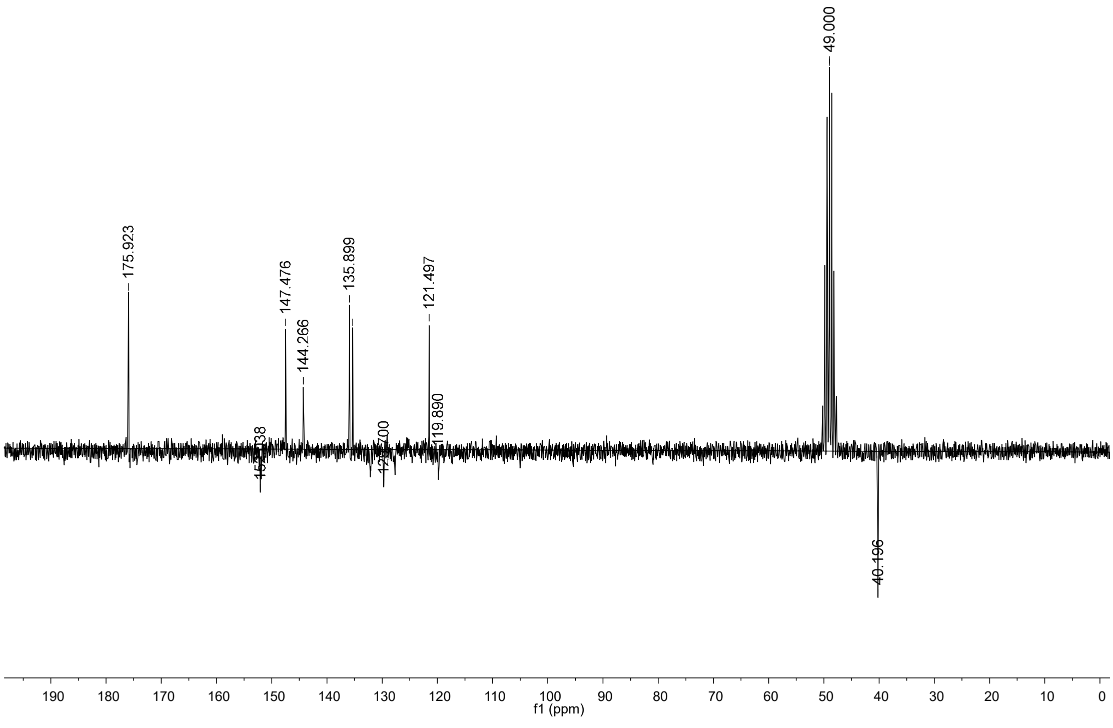

11. Copies of IR, ^{1}H NMR, ^{13}C NMR and HMBC spectra of product **11**

Figure S37. IR of **11**

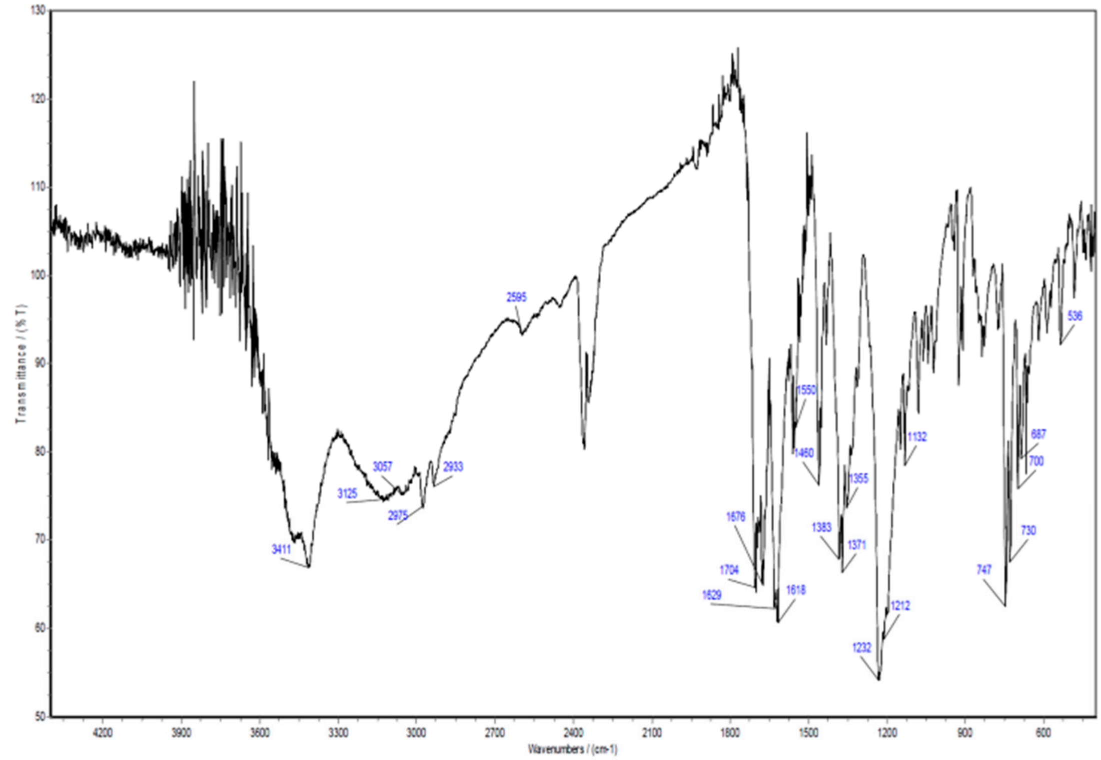

Figure S38. ^{1}H NMR of **11** (200 MHz, MeOD).

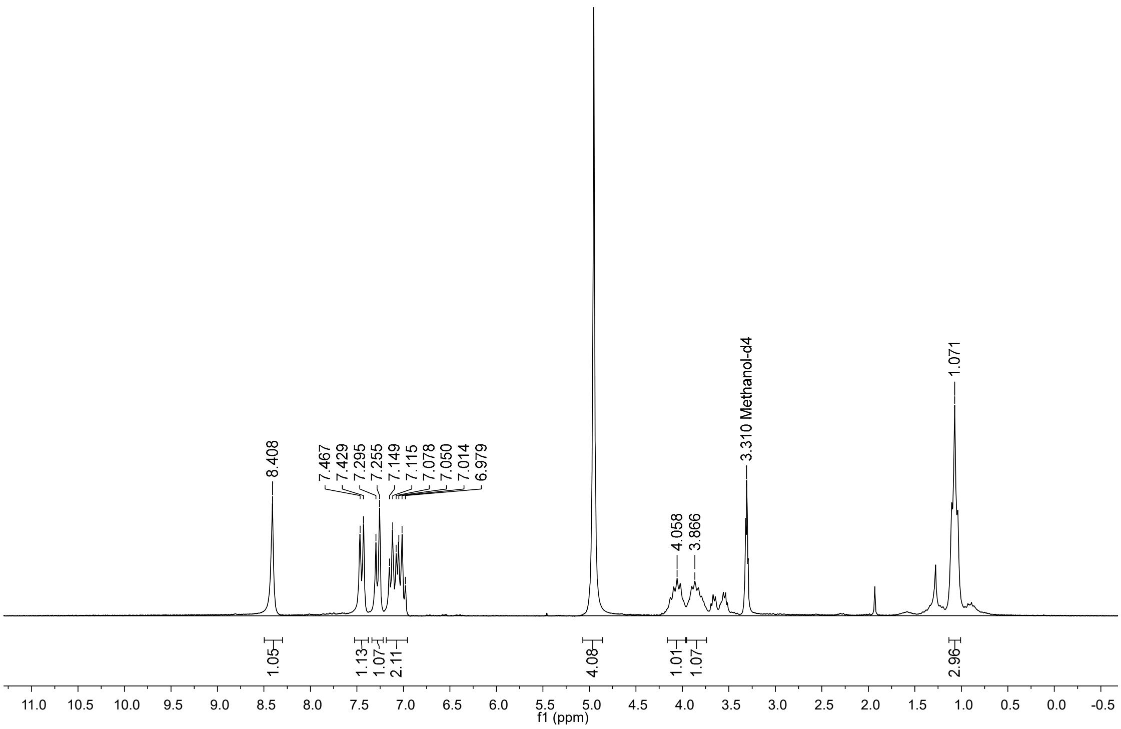

Figure S39. ^{13}C NMR of **11** (200 MHz, MeOD).

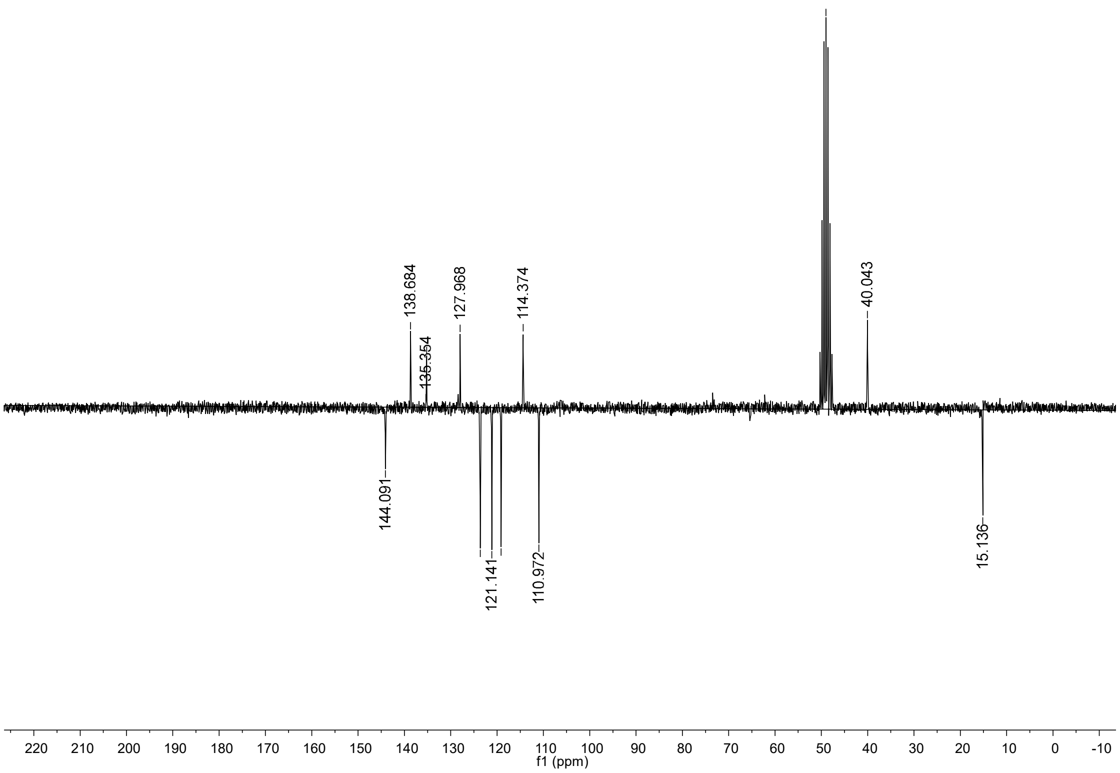

Figure S40. HMBC of **11** (500 MHz, MeOD).

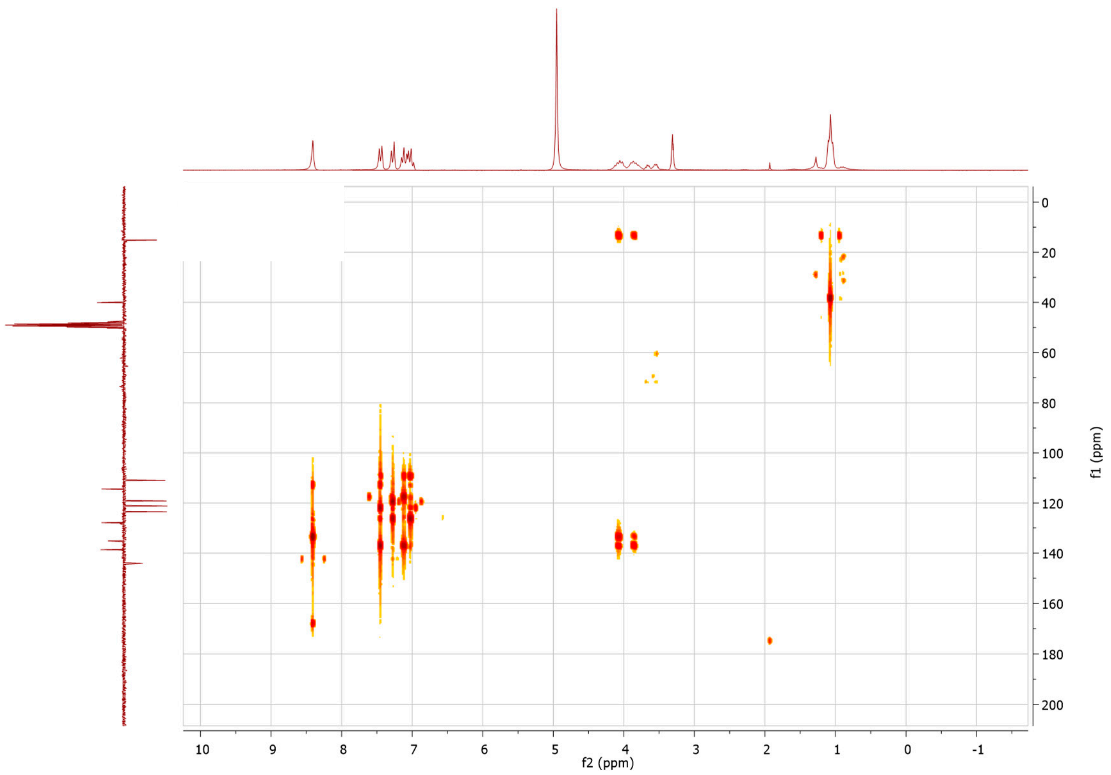

12. Copies of IR, ^{1}H and ^{13}C NMR spectra of product **12**

Figure S41. IR of **12**

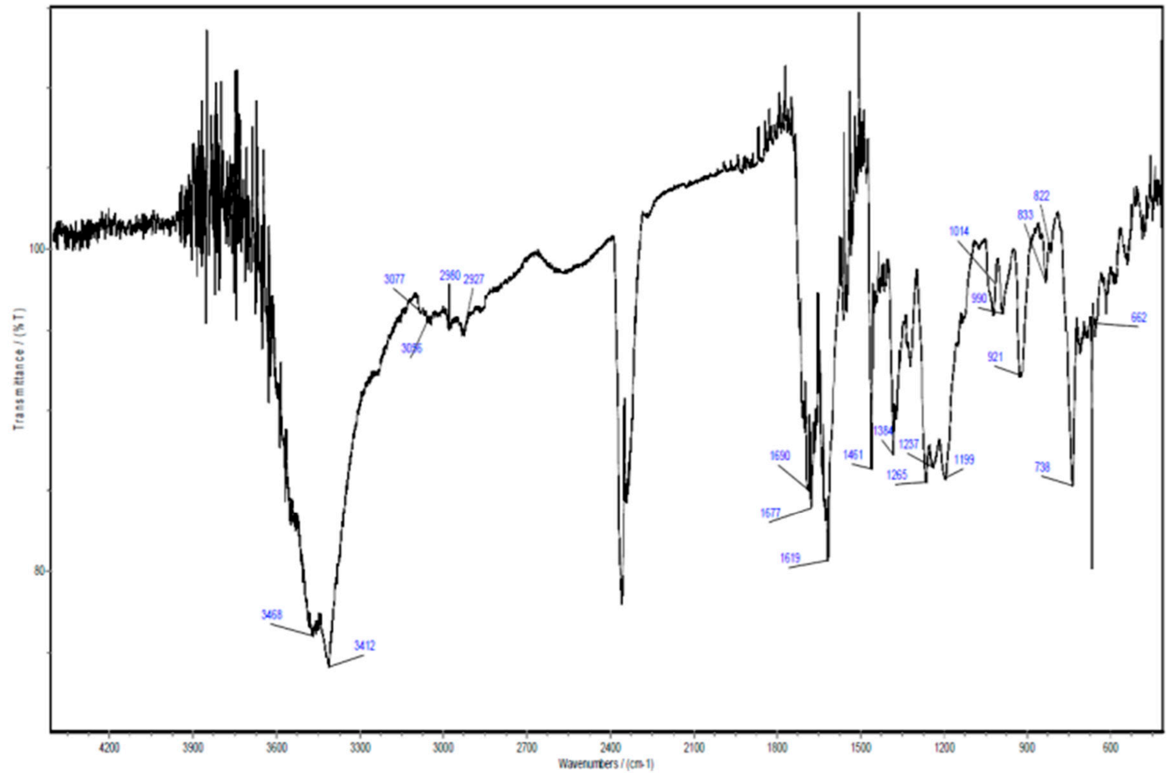

Figure S42. ^{1}H NMR of **12** (200 MHz, MeOD).

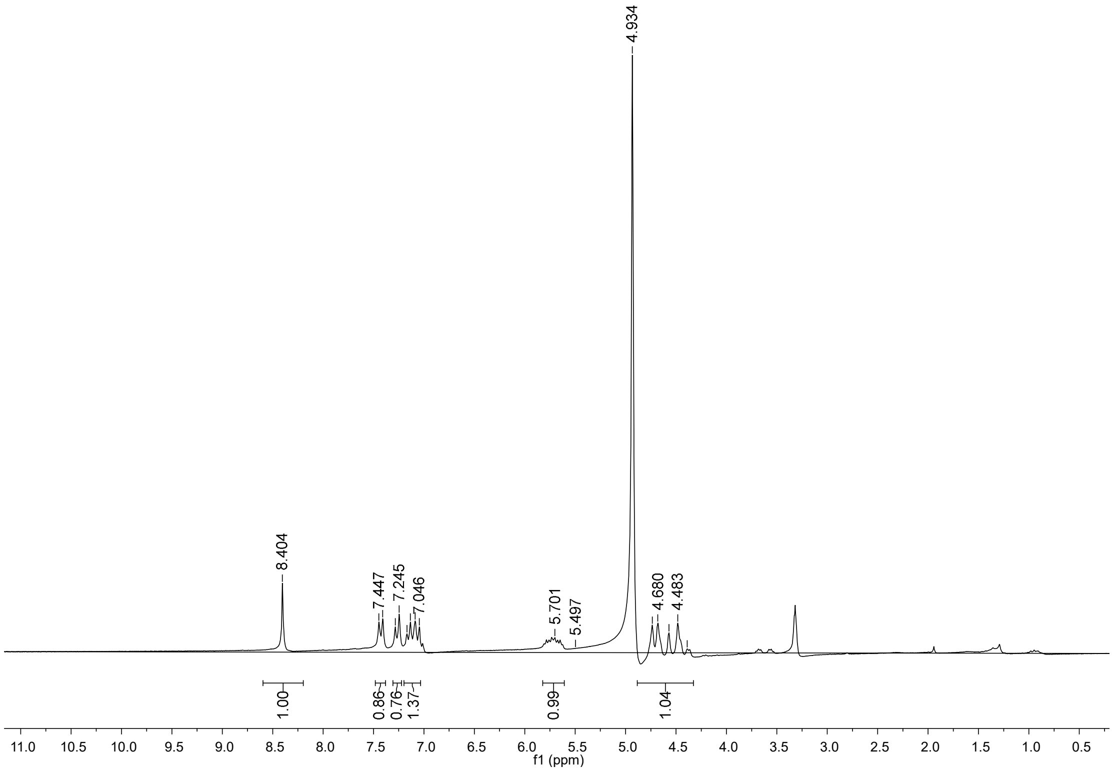

Figure S43. ^{13}C NMR of **12** (200 MHz, MeOD).

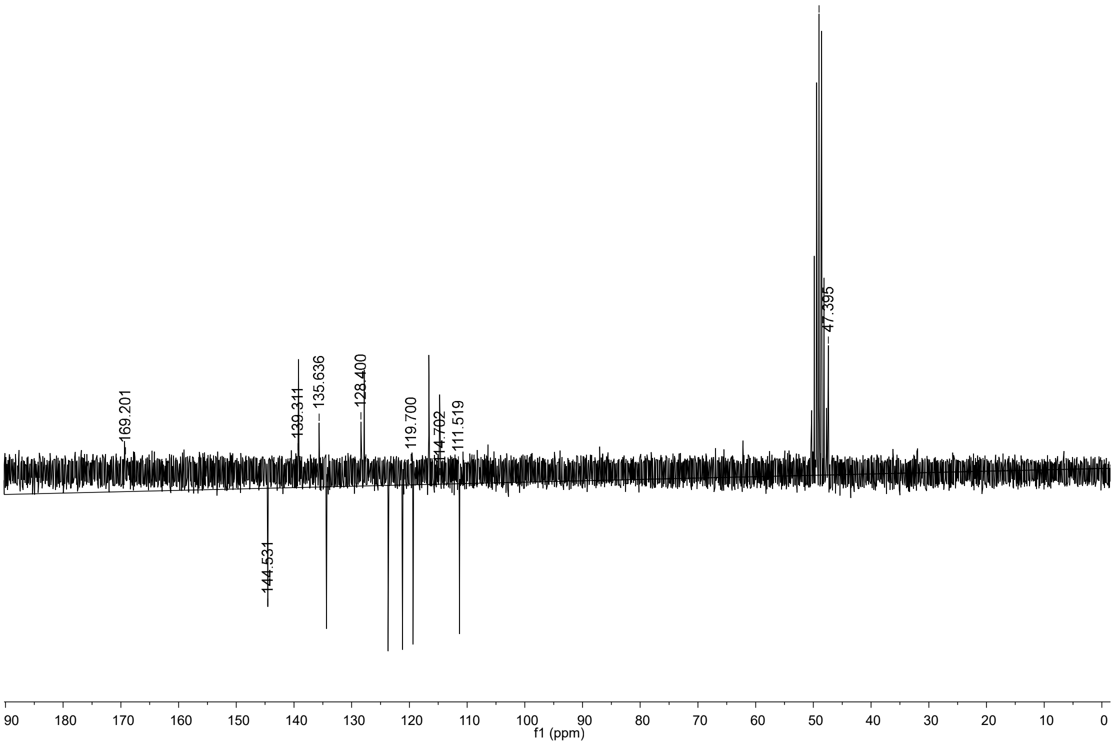

13. Copies of IR, ^{1}H NMR, ^{13}C NMR and HMBC spectra of product **13**

Figure S44. IR of **13**

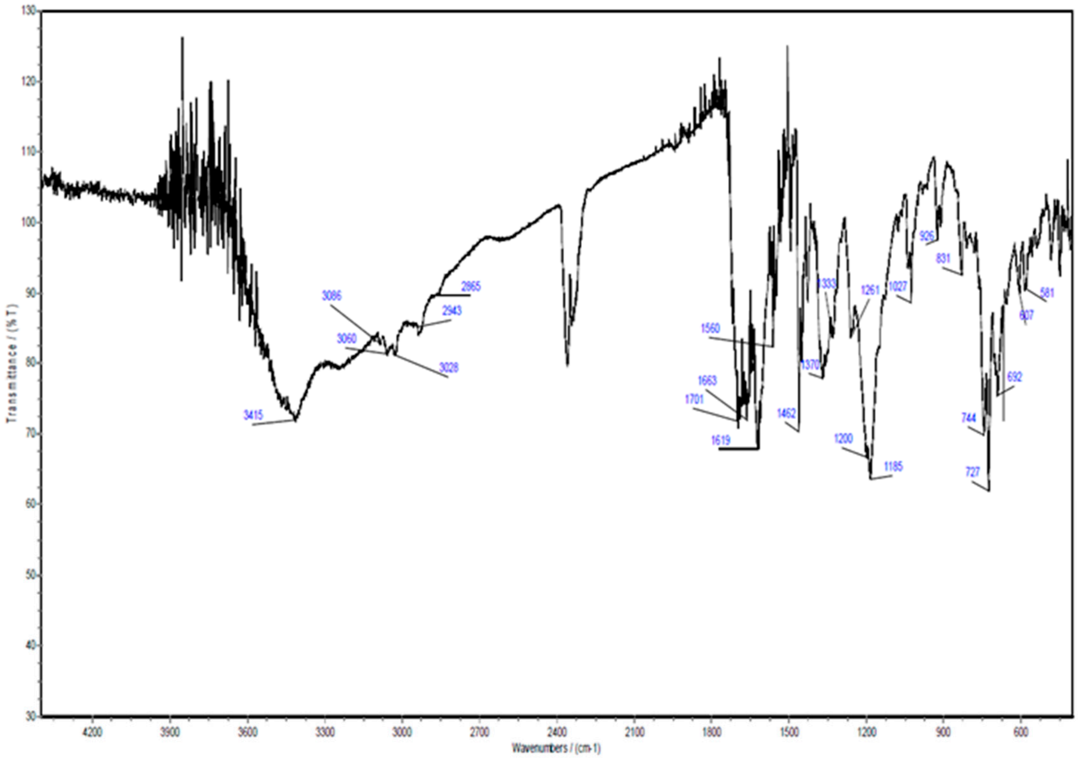

Figure S45. ^{1}H NMR of **13** (200 MHz, MeOD).

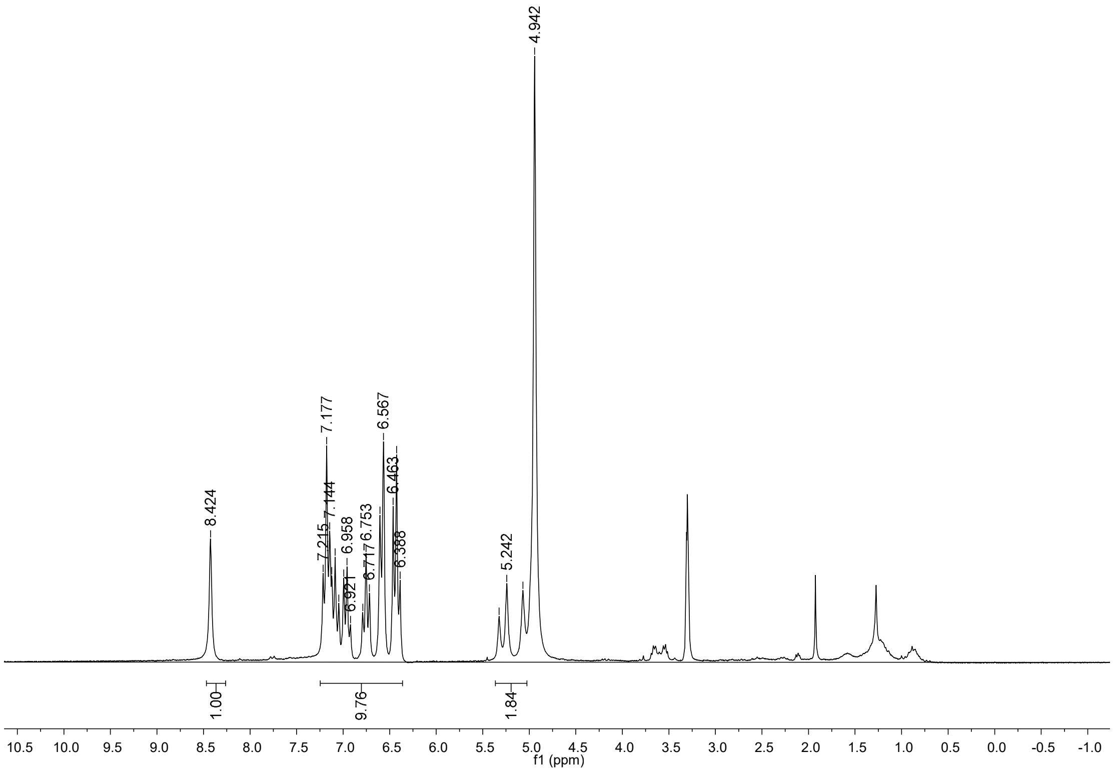

Figure S46. ^{13}C NMR of **13** (200 MHz, MeOD).

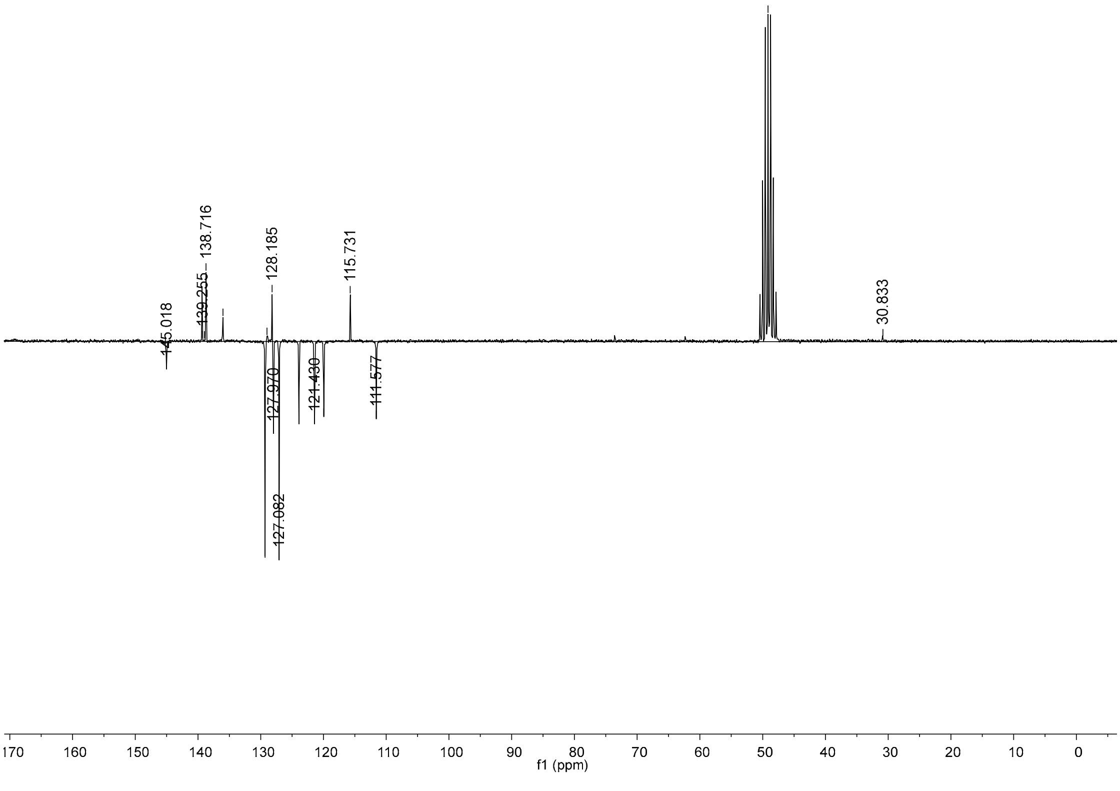

Figure S47. HMBC of **13** (500 MHz, MeOD).

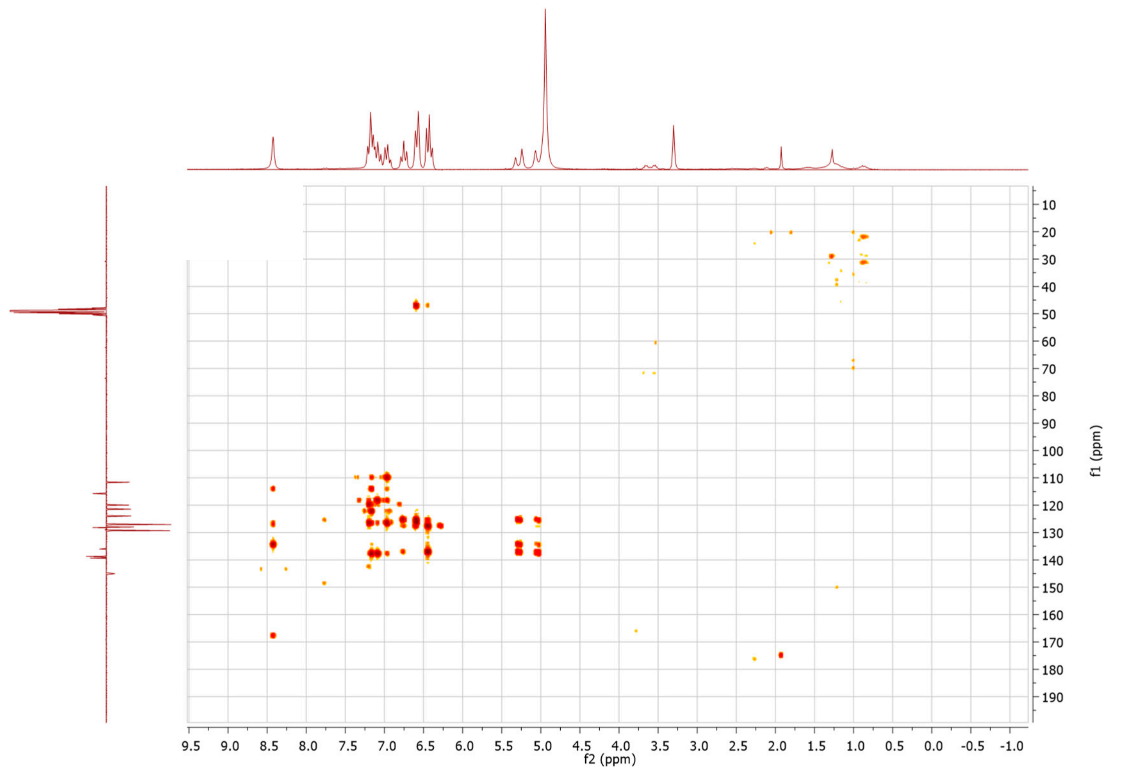

14. Copies of ^{1}H and ^{13}C NMR spectra of product **14**

Figure S48. ^{1}H NMR of **14** (200 MHz, $CDCl_3$).

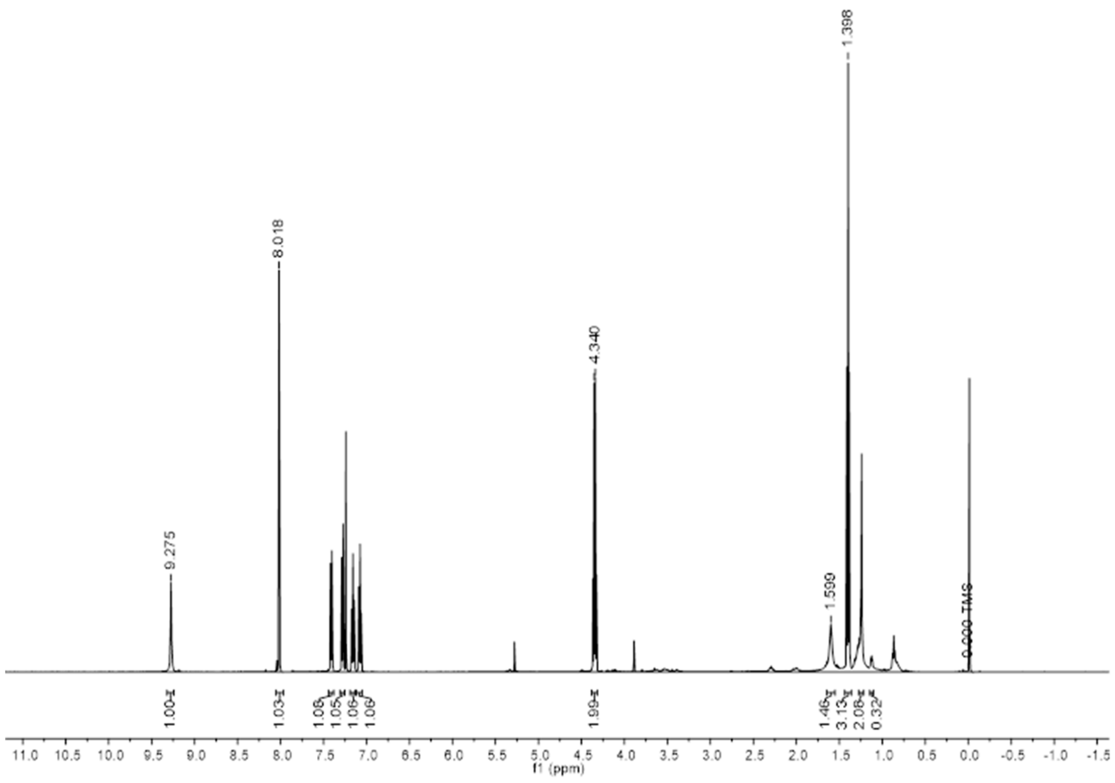

Figure S49. ^{13}C NMR of **14** (200 MHz, $CDCl_3$).

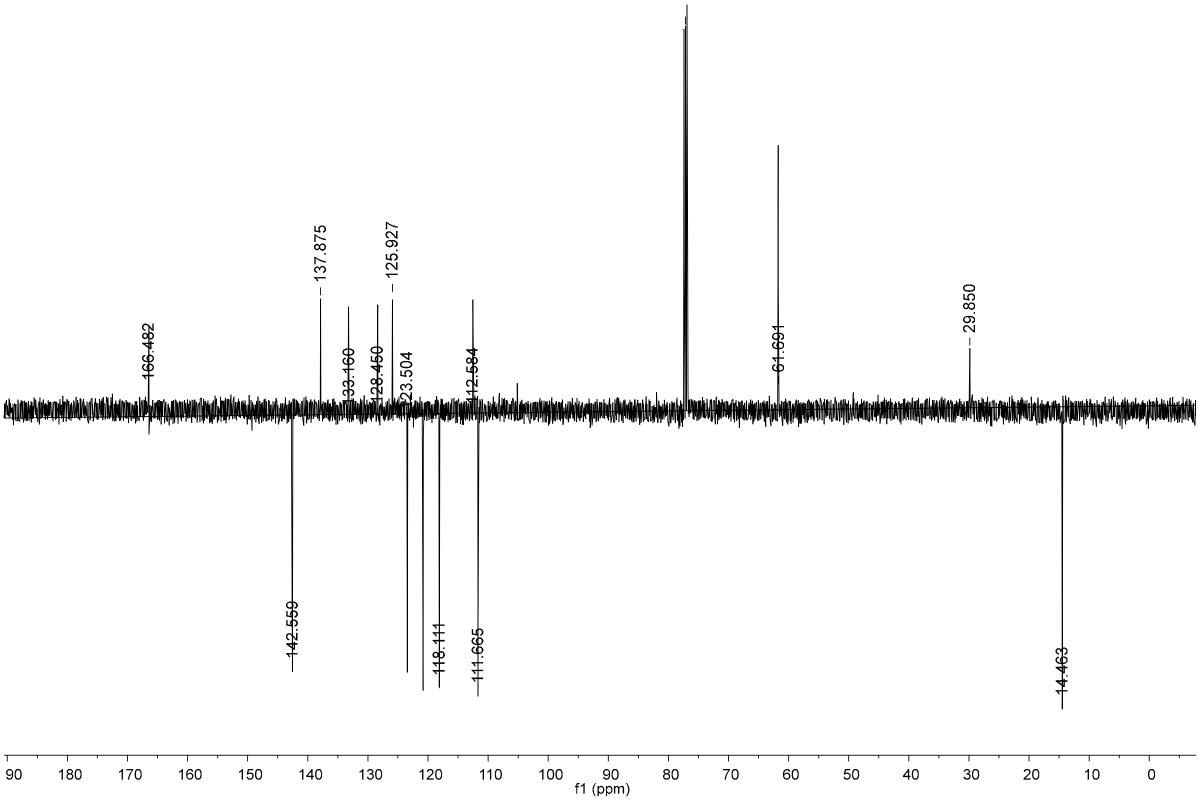

15. Copies of ^{1}H and ^{13}C NMR spectra of product **15**

Figure S50. ^{1}H NMR of **15** (200 MHz, $CDCl_3$).

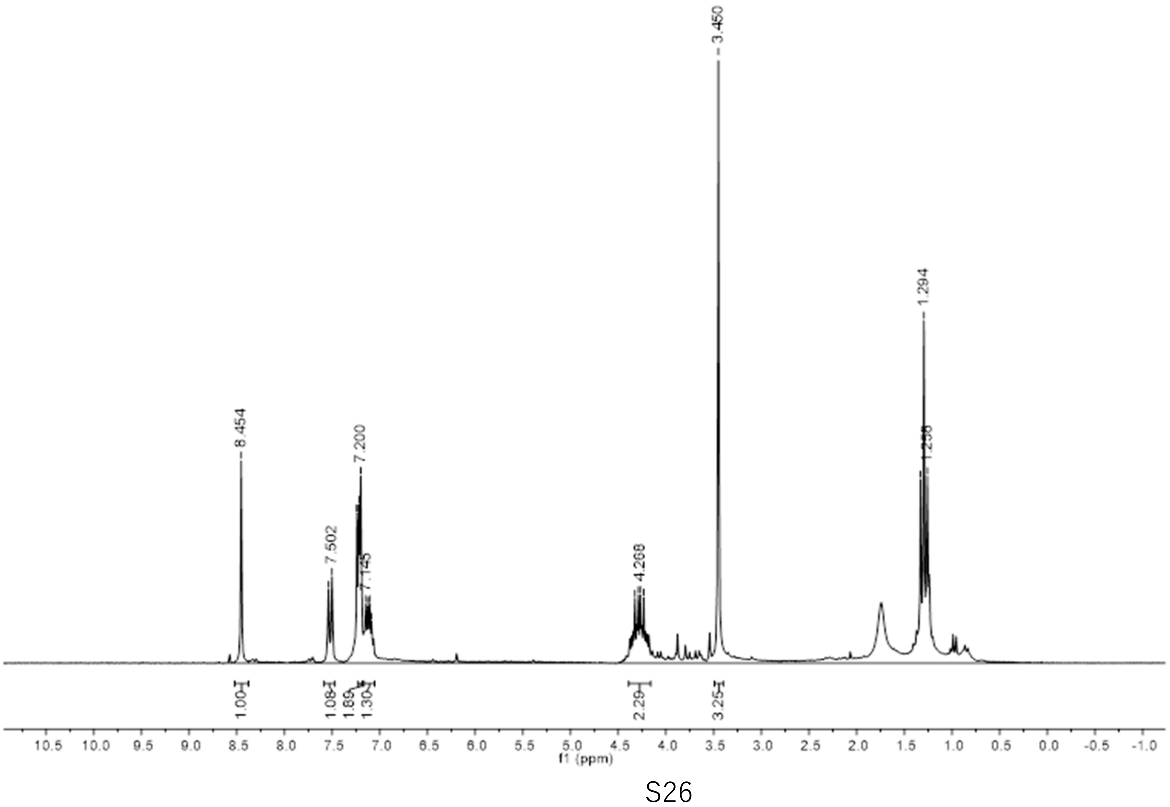

Figure S51. ^{13}C NMR of **15** (200 MHz, $CDCl_3$).

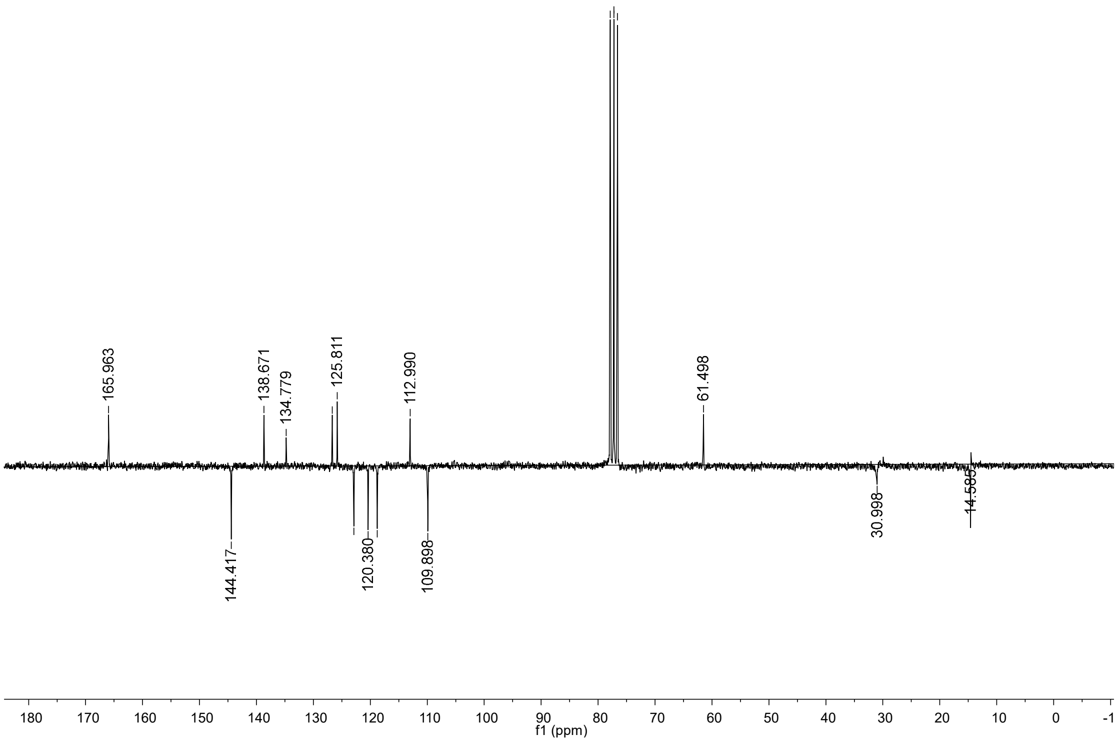

Supplement: Supplementary file 1 [file molecules-29-03859-s001.zip › molecules-3107252-supplementary.pdf]
